# Neural Networks Are Promising Tools for the Prediction of the Viscosity of Unsaturated Polyester Resins

**DOI:** 10.3389/fchem.2019.00375

**Published:** 2019-05-27

**Authors:** Julien Molina, Aurélie Laroche, Jean-Victor Richard, Anne-Sophie Schuller, Christian Rolando

**Affiliations:** ^1^Mäder Research, Mulhouse, France; ^2^Faculté des Sciences et Technologies, Université de Lille, USR 3290 MSAP, Miniaturisation pour l'Analyse, la Synthèse et la Protéomique, Villeneuve d'Ascq, France

**Keywords:** unsaturated polyester, viscosity, neural network, QSPR, hansen solubility parameters, prediction

## Abstract

Unsaturated polyester resins are widely used for the preparation of composite materials and fulfill the majority of practical requirements for industrial and domestic applications at low cost. These resins consist of a highly viscous polyester oligomer and a reactive diluent, which allows its process ability and its crosslinking. The viscosity of the initial polyester and the reactive diluent mixture is critical for practical applications. So far, these viscosities were determined by trial and error which implies a time-consuming succession of manipulations, to achieve the targeted viscosities. In this work, we developed a strategy for predicting the viscosities of unsaturated polyesters formulation based on neural networks. In a first step 15 unsaturated polyesters have been synthesized through high-temperature polycondensation using usual monomers. Experimental Hansen solubility parameters (HSP) were determined from solubility experiment with HSPiP software and glass transition temperatures (*T*_*g*_) were measured by Differential Scanning Calorimetry (DSC). Quantitative Structure—Property Relationship (QSPR) coupled to multiple linear regressions have been used to get a prediction of Hansen solubility parameters δ_d_, δ_*p*_, and δ_*h*_ from structural composition. A second QSPR regression has been done on glass transition temperature (prediction vs. experimental coefficient of determination *R*^2^ = 0.93) of these unsaturated polyesters. These unsaturated polyesters were next diluted in several solvents with different natures (ethers, esters, alcohol, aromatics for example) at different concentrations. Viscosities at room temperature of these polyesters in solution were finally measured in order to create a database of 220 entries with 7 descriptors (polyester molecular weight, *T*_*g*_, dispersity index Ð, polyester-solvent HSP RED, molar volume of the solvent, δ_*h*_ of the solvent, concentration of polyester in solvent). The QSPR method for predicting the viscosity from these 6 descriptors proved to be ineffective (*R*^2^ = 0.56) as viscosities exhibit non-linear phenomena. A Neural Network with an optimized number of 12 hidden neurons has been trained with 179 entries to predict the viscosity. A correlation between experimental and predicted viscosities based on 41 testing instances gave a correlation coefficient *R*^2^ of 0.88 and a predicted vs. measured slope of 0.98. Thanks to Neural Networks, new developments with eco-friendly reactive diluents can be accelerated.

## Introduction

Today composite materials find many applications in the fields of transport, construction as well as in sports and leisure (Biron, 2013). The unsaturated polyester resins used for the preparation of these composite materials have several advantages, mainly a favorable price ratio with respect to the mechanical and thermal properties (Mishra et al., 2003), good durability and a relatively good resistance to corrosion (Dagher et al., 2004), a low maintenance cost as well as good electrical, phonic and thermal insulation properties. It also lightens the structures compared to conventional metallic materials allowing to obtain better energy performances (Song et al., 2009). The investment cost related to machining composite materials by hand lay-up is also low (Biron, 2013).

The unsaturated polyesters are synthesized by high temperature polycondensation of diols with saturated and unsaturated diacids. The most used unsaturated monomers are maleic anhydride or fumaric acid. The water produced by the esterification reaction is eliminated by condensation in a Dean-Stark during the reaction. The number average molecular weight of the obtained polyesters are ~1,000 g.mol^−1^ (Fink, 2013). Depending on the monomers used in the polycondensation, the properties of polyester resins differ. For applications where the resin must be resistant to hydrolysis, monomers such as neopentyl glycol and isophthalic acid are particularly suitable. The use of diethylene glycol or dipropylene glycol makes possible to obtain flexible resins (Zaske and Goodman, 1998; Fink, 2013). Thus, there is a multitude of possible chemical structures depending on the intended application.

In order to be manipulated at room temperature and to be crosslinked, the polyesters are diluted in polymerizable solvents. The most commonly reactive diluent is styrene because it effectively reduces the viscosity of the unsaturated polyester in solution and efficiently copolymerizes with the fumarate units (Lewis and Mayo, 1948; Cousinet et al., 2015). However, styrene has been classified by the US Department of Health and Human Services as “reasonably anticipated to be a human carcinogen.” It is a very volatile monomer that has also been classified as a hazardous air pollutant by the US Environmental Protection Agency (Cousinet et al., 2015). In Europe, styrene has been classified as “reproductive toxicity category 2” by the European Chemicals Agency (ECHA). Methacrylate monomers are commonly used to replace styrene (Fink, 2013). However, monomers such as methyl, ethyl or butyl methacrylates have strong odors. This is a disadvantage for open mold applications. In addition, their reactivity ratio with fumarate units does not allow good crosslinking (Bengough et al., 1967). Many publications deal with the search for alternative reactive diluents, sometimes bio-sourced, in order to be able to eliminate styrene and to provide resins with less volatile and less toxic organic compounds (Sadler et al., 2012; Cousinet et al., 2014, 2015; Li et al., 2014; Dai et al., 2017; Panic et al., 2017; Yadav et al., 2018).

To develop a new resin, it is now necessary to multiply time-consuming manipulations. Firstly a polyester with a defined structure is synthesized, then diluted in a reactive solvent and finally crosslinked. The properties of the resin such as its viscosity at room temperature and its mechanical properties need to be measured for assessing its performance. Performing all of these steps take several days for a single try. The multitude of possible chemical structures as well as the diversification of available reactive diluents considerably extends the time required for the development of a new resin. The viscosity of polyester resins at room temperature is an important parameter to be respected in a specification. Indeed, the resin must be in a certain range of viscosity depending on its mode of application (Fink, 2013). Developing property prediction tools that use only theoretical values without manipulation is therefore a strategic issue, particularly in the industrial sector.

Neural networks are machine learning tools for connecting non-linear data with one or more target properties (Gasteiger and Zupan, 1993; Svozil et al., 1997). This type of algorithm has been used effectively in many scientific fields, especially in environmental or chemical applications (Behler, 2011; Torrecilla et al., 2013; Wei et al., 2016). Several studies have already been published on the prediction of polymer properties using neural networks, such as the glass transition temperature (Joyce et al., 1995; Mattioni and Jurs, 2002; Chen et al., 2008; Liu and Cao, 2009), intrinsic viscosity (Gharagheizi, 2007a) or lower critical solution temperature (Gharagheizi F., 2007b).

In this work, a neural network was set up in order to predict the viscosity of unsaturated polyester resins from simple descriptors. Once a polyester is synthesized, its number average molecular weight and its glass transition temperature are measured. The experimental Hansen solubility parameters (HSP) (Hansen, 2002) of the polyester are then obtained by solubilization of the polymer in 40 solvents followed by processing results on the HSPiP software (Abbott, 2013). Then, the polyester is solubilized by varying its concentration in solvents of different natures among those previously used. A database of 220 entries of polymer-solvent combination was set up including for the polyesters, their number average molecular weight, their glass transition temperatures and their Hansen parameters, for the solvents their molar volumes, their δ_*h*_ and the concentration of the polyester in solution. The resulting viscosity of the polyester in solution was measured with a rheometer for each entry. The neural network was subsequently optimized and trained with this database.

To be able to predict unsaturated polyester viscosity exclusively based on theoretical values without manipulation, the glass transition temperature as well as Hansen parameters of unsaturated polyesters have been correlated according to the theoretical chemical structure of the polyesters. Prediction methods have already been described in the literature for the glass transition temperature (Katritzky et al., 1996; Bicerano, 2002; Camacho-Zuñiga and Ruiz-Treviño, 2003; Krevelen and Nijenhuis, 2009) as well as the Hansen solubility parameters of polymers (Stefanis and Panayiotou, 2008; Krevelen and Nijenhuis, 2009). However, these methods generally relate to high average molecular weight polymers and are not necessarily adapted to unsaturated polyesters. In this work, a Quantitative Structure—Property Relationship (QSPR) method was applied to propose a simple method for determining the glass transition temperature and Hansen solubility parameters for unsaturated polyesters. The experimental values used in the neural network can be replaced in the future by the predicted values obtained by QSPR.

Data capitalization and processing has become a strategic topic for predicting phenomena (Dong et al., 1996; Zhang et al., 1998; Marengo et al., 2004). Being able to predict the viscosity of polyester resins to see if they fulfill specifications and minimize the number of tests is undoubtedly of high added value for thermoset resins industrial companies. Today, the establishment of a machine learning system has become more accessible, so its use in chemical companies will certainly grow in the coming years.

## Materials and Methods

### Reagents

Propylene glycol (PG), dipropylene glycol (DPG), neopentyl glycol (NPG), cyclohexanedimethanol also known as 1,4-bis(hydroxymethyl)cyclohexane (CHDM), 2-ethylhexanol (EH), benzyl alcohol (AB), maleic anhydride (AM), itaconic acid (IT), fumaric acid (AF), phthalic anhydride (PA), adipic acid (AA) were provided by the Mäder group. They were used as received without further purification.

All solvents used for the determination of Hansen parameters are laboratory grade and were used as received without further purification.

### Synthesis of the Prepolymer

The prepolymer was synthesized by the melt polycondensation between diols and diacids. The components were mixed in a 1 L four-necked round-bottom flask connected with a stirrer, a temperature probe connected to the heater, a Dean–Stark, and a N_2_ gas inlet. No catalyst was used in this work. The reaction was carried out at a temperature of 200°C under a nitrogen atmosphere. The reaction was carried out until the acid value reached 30. The acid value (AV) is defined as the number of milligrams of KOH needed to neutralize 1 g of resin and was measured according to ASTM D465-01. Around 1 g of resins was titrated with a KOH solution in isopropanol (0.1 M).

### Prepolymer Characterization

The size exclusion chromatography (SEC) used was a Shimadzu Prominence fitted with a Refractive Index (RI) detector (RID-20A) and an UV detector (SPD-20A). The columns (KF-802 and KF-803L from Shodex) were eluted with tetrahydrofuran (THF) at a flow rate of 1 mL/min at 30°C. The samples were previously prepared by dissolving 10 mg of sample in 1 mL THF. The solution was then filtered through a PTFE filter with a pore diameter of 0.45 μm. A volume of 20 μL was injected into the size exclusion chromatography to carry out the analysis. The SEC has been calibrated with poly(styrene) standards. The number average molecular weights were determined from the UV detector absorbance.

The glass transition temperature (*T*_*g*_) of the prepolymers was measured by differential scanning calorimetry, DSC, using a Q20 TA Instruments in hermetic aluminum capsules with a scan rate of 10°C/min from −80°C to 150°C under N_2_ (50 mL/min). The second heating run was used to determine the *T*_*g*_ with the TA Instruments software.

### Hansen Solubility Parameter Experimental Determination

The solubility of the polymers was assessed by dissolving 100 mg in 1 mL of solvent at room temperature. Solubility was assessed after 24 h of agitation using a Vortex-Genie 2 from Scientific Industries. The 40 solvents tested were acetic acid, acetone, acetonitrile, aniline, benzonitrile, benzyl alcohol, γ-butyrolactone, m-cresol, cyclohexane, cyclohexanone, o-dichlorobenzene, diethylene glycol, dimethyl formamide, 1,4-dioxane, ethanol, ethyl acetate, ethylene glycol, ethylene glycol monomethyl ether, formamide, formic acid, furan, hexane, isobutyl alcohol, methanol, methyl ethyl ketone, *N*-methyl formamide, methyl methacrylate, *N*-methyl-2-pyrrolidone, methylene dichloride, morpholine, nitrobenzene, 1-pentanol, 1-propanol, propionitrile, propylene carbonate, propylene glycol monomethyl ether, styrene, tetrahydrofuran, toluene, water (Delgove et al., 2017). The Hansen solubility parameters δ_*d*_, δ_*p*_, δ_*h*_ and the solubility sphere radius *R*_0_ of the unsaturated polyesters were obtained using the HSPiP software. A sphere centered on the HSP of the polyester and radius *R*_0_ constitutes the sphere of solubility of the polyester. Solvents whose HSP are inside the sphere allow the solubilization of the polyester. The polyester is insoluble in solvents having HSP outside the sphere.

### Unsaturated Polyester—Solvent Compatibility Determination

Once the HSP of the polyesters were obtained, the compatibility of each polyester in solvents of different natures was quantified. Firstly, the distance *R*_*a*_ in a three-dimensional space between the Hansen parameters of the polyester (P) and the Hansen parameters of the solvent (S) was calculated using the Equation (1) (Krevelen and Nijenhuis, 2009).

(1) $$\begin{array}{r} R_{a}^{2}=4.0\times {\left(\delta_{dP}-\;\delta_{dS}\right)}^{2}+{\left(\delta_{pP}-\delta_{pS}\right)}^{2}+{\left(\delta_{hP}-\delta_{hS}\right)}^{2} \end{array}$$

The Relative Energy Difference (*RED*) was then calculated by performing the ratio of *R*_*a*_ to *R*_0_ (Equation 2) corresponding to the solubility radius of the unsaturated polyester (Krevelen and Nijenhuis, 2009).

(2) $$\begin{array}{r} RED=\;\frac{R_{a}}{R_{0}} \end{array}$$

Thus, the *RED* gives a simple numerical value for characterizing the compatibility of a polymer in a solvent. According to Hansen's theory, two compounds are very compatible if their *RED* approaches 0 because their Hansen solubility parameters are very close. If their RED is equal to 1, it means that the polyester is at the limit of solubility in the solvent and therefore almost incompatible. A *RED* >1 means that the polyester is not soluble in the solvent tested (Krevelen and Nijenhuis, 2009).

### Creation of the Polyester Resin Database

In order to develop the database, the unsaturated polyesters synthesized were diluted in various solvents among those used in Part 2.3 and at different concentrations. Apparent viscosities were measured at 23°C as a function of shear rate over the range 1–100 s^−1^ using the viscometry function of a controlled stress and strain rheometer (Anton Paar MCR 301). A parallel plate geometry has been used with a diameter plate of 25 mm (PP25) and a gap of 1 mm.

The database contains 220 entries including for each of them the number average molecular weight of the polyester *M*_*n*_ (obtained by SEC), its index polydispersity Ð, and its glass transition temperature *T*_*g*_ (obtained by DSC), the *RED* polymer-solvent compatibility (obtained via HSPiP), the molar volume of the solvent *M*_*vol*_(obtained via HSPiP), the concentration of the polyester in the solution and the measured viscosity at 23°C of the polyester in solution. This database is provided in Table S1.

### QSPR Modeling With Multiple Linear Regression (MLR)

Quantitative Structure—Property Relationship (QSPR) modelizations were carried out by multiple linear regression. Different descriptors *x*_*i*_ are correlated with one or more responses. The linear relation linking the descriptors to this response is given in Equation 3.

(3) $$\begin{array}{r} y=a_{0}+a_{1}x_{1}+a_{2}x_{2}+\ldots +a_{i}x_{i}+e \end{array}$$

The values *a*_*i*_ are the regression coefficients. The purpose of multiple linear regression is to determine the value of these coefficients by the least squares method. These modelizations were realized with the software Cosmoquick version 1.7 (COSMOlogic, Leverkusen, Germany) (Loschen and Klamt, 2012).

### Artificial Neural Network

Neural networks are a type of machine learning tool which link several input data with output data by non-linear relations (Gasteiger and Zupan, 1993; Svozil et al., 1997). They present a real advantage over conventional linear mathematical approaches (Díaz-Rodríguez et al., 2014; Cancilla et al., 2016). The use of neural networks allows to find physico-chemical models already described in the literature or even to discover original models (Behler, 2011; Díaz-Rodríguez et al., 2015).

A neural network is divided into several layers, each composed of neurons and interconnected by synapses (Díaz-Rodríguez et al., 2014). The first layer, called the input layer, introduces into the neural network the values of the different descriptors influencing the target property at the output of the neural network. In this study, several physicochemical data describing both the polyesters as well as the solvents properties were used in this input layer.

The second part of the neural network is the hidden learning layer. It contains neurons that allow non-linear calculations to obtain the relationship between input and output data (Gasteiger and Zupan, 1993; Cancilla et al., 2014a,b). Each learning neuron performs a linear combination of input data multiplied by the weight of the synapses associated with that data. An additional constant, called bias, is added to this linear combination in order to add an extra degree of freedom to the neural network to better match input and output data. A function that can be linear or not transforms the value obtained in order to obtain the output signal of the neuron. The most common non-linear functions are the hyberbolic tangent or the sigmoid. A multitude of other activations functions exist and research are still on-going on the development of new functions (Xu et al., 2015). This output value is then introduced as an input value for the next layer of neurons.

The number of neurons in the hidden layer must be optimized in order to have the best learning and to get the best prediction accuracy. A low number of learning neurons will tend to limit the learning ability of complex problems by the neural network whereas an excessive number of neurons can lead to an over-fit of prediction and an increase in the gap compared to the experimental target values. Although different rules emerge to fix the number of hidden neurons based on the number of input and output data, it is also possible to test the evolution of the prediction error with respect to the experimental one by changing the number of learning neurons (Sheela and Deepa, 2013). In the initial state, values of the synapses weights are fixed randomly. The training protocol is based on an algorithm seeking to reduce the difference between the experimental target values compared to the values predicted by successive iterations that modify the weight of the synapses. There are different types of training algorithms, each of which is more suitable for a kind of applications (Torrecilla et al., 2008). A neural network can continue the iterations until the predicted values fit perfectly with the training data. However, this can cause over-fit due to the consideration of non-general trends such as experimental errors or noise. Verification of the reliability of the neural network can be performed with a set of data that have not been used for the modification of synaptic weights during training (Cancilla et al., 2014a). When the error between experimental values and predicted values begins to increase, it means that the training phase has undergone too many iterations.

Neural designer desktop version 2.9.5 (Artelnics, Salamanca, Spain) has been employed for the neural network design and its optimization.

## Results and Discussion

### Unsaturated Polyesters Synthesis

Fifteen unsaturated polyesters have been synthesized from the monomers conventionally used in industry. The stoichiometric ratio between the reagents called *r* corresponds to the initial molar amount of carboxylic acid groups on the initial molar amount of alcohol groups provided by the diacids and glycols of the polycondensation reaction. These different structures are listed in Table 1. They were characterized initially by DSC and SEC in order to obtain the glass transition temperature *T*_*g*_, the number average molecular weight *M*_*n*_ and the dispersity index Ð.

**Table 1:** Structures of the unsaturated polyesters synthesized.

**Polyester**	**Monomer 1 (mol%)**	**Monomer 2 (mol%)**	**Monomer 3 (mol%)**	**Monomer 4 (mol%)**	**Monomer 5 (mol%)**	***r***	***T_g_(°C)***	***M_*n*_* (g/mol)**	**Ð**
**1**	PG 80%	DPG 20%	AM 67%	AP 27%	AA 6%	0.97^a^	3.9	1,880	3.90
**2**	NPG 70%	PG 30%	–	AF 100%	/	0.93	9.1	2,678	2.23
**3**	NPG 70%	PG 30%	–	AM 60%	AP 40%	0.90	16.3	1,560	2.13
**4**	NPG 70%	PG 30%	–	AM 50%	AP 50%	0.90	24.4	1,652	2.51
**5**	NPG 70%	PG 30%	–	AM 70%	AP 30%	0.90	11.7	1,640	1.90
**6**	NPG 70%	PG 30%	–	AM 60%	AP 40%	0.91	16.0	1,780	1.87
**7**	NPG 50%	PG 50%	–	AM 60%	AP 40%	0.96	20.1	2,530	2.90
**8**	NPG 70%	PG 30%	–	IT 60%	AP 40%	0.98	12.1	1,205	2.64
**9**	PG 100%	–	–	AM 60%	AP 40%	0.90	22.0	1,610	3.69
**10**	PG 100%	–	–	AM 60%	AP 40%	0.91	23.6	1,760	1.5
**11**	NPG 70%	PG 30%	EH 5%	AM 60%	AP 40%	0.94	2.4	1,220	2.09
**12**	NPG 70%	PG 30%	–	AM 60%	AP 40%	0.96	21.2	1,960	2.59
**13**	CHDM 70%	PG 30%	–	AF 60%	AP 40%	0.92	22.6	2,420	1.84
**14**	DPG 100%	–	–	AF 60%	AP 40%	0.92	−6.5	1,409	2.47
**15**	DPG 50%	NPG 50%	–	AF 60%	AP 40%	0.91	1	1,330	2.20
**16**	NPG 70%	PG 30%	–	AM 60%	AP 40%	0.75	−2.5	950	1.84
**17**	NPG 70%	PG 30%	–	AM 60%	AP 40%	0.93	20.9	2,090	2.70
**18**	CHDM 100%	–	–	AF 60%	AP 40%	0.92	29.4	1,995	2.21
**19**	NPG 70%	PG 30%	AB 5%	AM 60%	AP 40%	0.94	11.2	1,410	2.23
**20**	NPG 30%	CHDM 70%	/	AF 60%	AP 40%	0.92	23.5	1,760	2.16
**21**	NPG 70%	PG 30%	/	AF 60%	AA 40%	0.9	−20.7	1,350	2.38

a *Final acid number = 50 mgKOH/g (instead of 30 mgKOH/g)*.

During the reaction, the maleate units are isomerized into fumarate units. However, the isomerization rate depends mainly on the monomer composition of the resin (Curtis et al., 1964). Diols with secondary alcohols such as propylene glycol promote isomerization in contrast to diols having only primary alcohols. The presence of phthalic anhydride also promotes isomerization. Maleate units (*Z-*double bond) do not have the same properties as fumarate units (*E*-double bond) (Ebewele, 2000; Krevelen and Nijenhuis, 2009). In order to minimize the presence of maleates in the reaction, fumaric acid has been used in syntheses with primary diols or without phthalic anhydride.

The glass transition temperature *T*_*g*_ of the polyesters depends on the structure of the monomers used during the synthesis as well as the final average molecular weight obtained. The introduction of monomers comprising ether bridges such as dipropylene glycol or diethylene glycol allows the flexibilization of the polyester chains and therefore the lowering of the glass transition temperature of the polyesters (Young and Lovell, 1996; Zaske and Goodman, 1998; Ebewele, 2000). In order to be able to compare the impact of these monomers on the glass transition temperature, the acid monomer composition as well as the targeted degree of polymerization was fixed for polyesters described in polyesters **3**, **14**, and **15**. The polyester **4** composed solely of dipropylene glycol has a *T*_*g*_ of −6.5°C whereas the polyester **15** comprising 50% of neopentyl glycol and 50% of dipropylene glycol has a *T*_*g*_ of 1°C. A polyester without ethers monomers such as the one described in polyester **3** has a higher *T*_*g*_ of 16.3°C. The use of aromatic monomers such as orthophthalic anhydride also modulate the glass transition temperature of the unsaturated polyesters (Zaske and Goodman, 1998; Ebewele, 2000). The degree of polymerization as well as the glycol composition of the polyesters described in polyester **4**-**6** are similar while the ratio of maleic anhydride to orthophthalic anhydride has been varied. The increase in the ratio in favor of orthophthalic anhydride within the polyester induces an increase in the glass transition temperature. On the contrary, the introduction of long aliphatic chain within the polyester has a plasticizing action and thus induces a decrease in the glass transition temperature (Young and Lovell, 1996; Zaske and Goodman, 1998; Ebewele, 2000). When the orthophthalic anhydride is replaced by adipic acid, which has an aliphatic chain, the glass transition temperature drastically decreases (polyester **21**: *T*_*g*_ = −20.7°C vs. polyester **3**: *T*_*g*_ = 16.3°C). In the same way, the incorporation of a mono-functional aliphatic alcohol such as 2-ethylhexanol has a plasticizing action and a decrease in the glass transition temperature is observed (polyester **11**: *T*_*g*_ = 2.4°C vs. polyester **3**: *T*_*g*_ = 16.3°C).

The use of branched monomers such as neopentyl glycol or propylene glycol induces a steric hindrance and thus restricts the polymer chain rotation (Young and Lovell, 1996; Ebewele, 2000). Neopentyl glycol also has a symmetry with its two CH_3_ groups in comparison to propylene glycol which has only one CH_3_ group. Despite a larger steric hindrance, this symmetry induces a drop in the glass transition temperature (Mark, 2007). Moreover, neopentyl glycol has an additional CH_2_ group relative to propylene glycol which makes the polyester more flexible. The polyester **9** composed solely of propylene glycol for the glycol portion has a glass transition temperature of 22.0°C. When 70 mol% of propylene glycol is replaced by neopentyl glycol (polyester **3**), the glass transition temperature decreases to 16.3°C. The introduction of cycloaliphatic monomers such as cyclohexanedimethanol, for example, stiffens the polyester chains (Turner et al., 2001). The replacement of propylene glycol of polyester **3** by cyclohexanedimethanol involves an increase in the glass transition temperature (**20** *T*_*g*_ = 23.5°C vs. **3** *T*_*g*_ = 16.3 °C). The polyester **18** containing only cyclohexanedimethanol has a glass transition temperature of 29.4°C. The influence of the number average molecular weight of the polyester was also studied. The monomer composition of the polyesters **3**, **16**, **17** was kept constant while varying the molecular weight. Obviously, the glass transition temperature increases as the average molecular weight of the polymer increases (Ebewele, 2000; Mark, 2007).

### Hansen Solubility Parameter Experimental Determination

In order to predict the solution viscosity of a polyester, it is important to know its compatibility with different types of solvent (Flory, 1942; Hillyer and Leonard, 1973; Young and Lovell, 1996). Indeed, a polyester containing a large number of polar groups adopt a different behavior in an apolar solvent (i.e., xylene) or in a polar solvent (i.e., water or ethanol). The Hansen solubility parameters (Krevelen and Nijenhuis, 2009) were therefore measured in order to be able to compare them with the solubility parameters of the various solvents subsequently tested for the prediction of viscosities. The measured parameters are listed in Table 2.

**Table 2:** Hansen solubility parameter of the synthesized unsaturated polyesters.

**Polyester**	***δ_*d*_***	***δ_*p*_***	***δ_*h*_***	***δ***	***R*_0_**
**1**	16.6	14.2	3.9	22.1	13.1
**2**	19.0	9.2	8.5	21.0	6.0
**3**	17.8	13.4	4.4	22.7	12.7
**4**	18.7	14.6	5.1	24.3	13.6
**5**	17.8	13.5	4.4	22.7	12.7
**6**	18.8	12.8	5.8	23.5	12.1
**7**	18.8	13.7	5.4	23.9	12.9
**8**	17.7	13.5	4.4	22.7	12.7
**9**	17.5	13.7	4.5	22.7	12.5
**10**	18.0	13.2	5.9	23.1	11.6
**11**	17.5	13.8	4.4	22.6	12.6
**12**	18.7	14.6	5.1	24.2	13.5
**13**	19.4	7.0	7.8	22.1	8.6
**14**	17.3	13.6	4.0	22.4	12.9
**15**	17.7	13.5	4.4	22.7	12.7
**16**	17.2	11.7	6.9	21.9	11.5
**17**	18.1	13.2	5.1	23.0	12.3
**18**	19.1	6.7	7.4	21.5	6.6
**19**	17.4	13.8	4.4	22.6	12.6
**20**	17.9	8.0	8.5	21.4	8.7
**21**	18.7	13.4	5.1	23.6	12.6
Average	18.1	12.4	5.5	22.7	11.6
Standard deviation	0.7	2.4	1.4	0.9	2.14

The δ_*d*_ of the 21 unsaturated polyesters synthesized, does not seem to be influenced by the variation of the monomers used. The standard deviation is low compared to the average of measured δ_*d*_. Polyesters **13** and **18** have the highest δ_*d*_ (19.4 and 19.1 MPa^1/2^). Both of these polyesters have cyclohexanedimethanol units within their chains. The polyester **13** has 70 mol% of cyclohexanedimethanol relative to total glycols while polyester **18** is composed of 100% cyclohexanedimethanol. These cycloaliphatic units have a high density of carbon relative to other glycols which induces the high value of δ_*d*_. The number average molecular weight of polyesters has an influence on δ_*d*_. The higher the number average molecular weight, the more δ_*d*_ increases. This can be explained by the fact that an increase in the number of average units in the polyester gives rise to a lesser importance of the functions allowing the hydrogen bonds (alcohols or terminal acids) with respect to the aliphatic functions.

The different δ_*p*_ measured have an average of 12.4 MPa^1/2^ with a standard deviation of 2.4 MPa^1/2^. There is therefore a greater variation compared to the δ_*d*_ of the different polyesters. Polyesters **1**, **4**, **12** have the highest δ_*p*_ values with respective values of 14.2, 14.6, 14.6 MPa^1/2^. They also have the greatest number of functional groups CH and quaternary C compared to other polyesters. These two types of groups induce asymmetries as well as an increase of the rigidity of the polyesters. These functional groups prevent the packing of the polyester chains by the irregularities they create within the polyester chain (Ebewele, 2000).

Polyesters **2**, **13**, **18**, and **20** have the lowest δ_*p*_. Firstly polyester **2** has a structure composed only of maleate/fumarate units for the acid part. This singularity increases the regularity of the polyester chain with respect to a maleate/aromatic mixture. This regularity brings the polyester chains closer together. It is also composed mainly of neopentyl glycol which does not have asymmetric carbons. The polyesters **13**, **18**, and **20** have a high content of cyclohexanedimethanol at the origin of the low δ_*p*_. The cyclohexanedimethanol do not have asymmetry centers and are therefore more regular than typical propylene glycol units (Turner et al., 2001).

The variation of δ_*h*_ is more important. It has indeed a significant standard deviation (1.4) with respect to its average of 5.5 for the 21 unsaturated polyesters. Polyesters **2**, **13**, **18**, and **20** which have structures without asymmetric functions also have the highest values of δ_*h*_. However, these four resins also have the lowest *R*_0_ of all the polyesters. They have the spheres of the smallest solubilities and are therefore soluble in less solvents than other polyesters (Krevelen and Nijenhuis, 2009). A small solubility radius indicates that the polyester prefers to create inter-molecular bonds instead of bonding with the solvent in which it is in solution. In order to be able to create inter-molecular bonds, however, the polyester must be regular and free of asymmetric functions so that the chains are close to one another (Young and Lovell, 1996; Ebewele, 2000; Delgove et al., 2017). This proximity allows the establishment of inter-molecular links. On the contrary, if the polyesters have many asymmetric functions, the polyester chains will not be able to get closer. Solvent molecules can thus more easily establish interactions with the polymer chains. The cyclohexanedimethanol unit does not have asymmetric functions. In polyester **13**, **18**, and **20** chains, it allows the packing of the chains and thus the lowering of the radius of the solubility sphere. Polyesters which possess a large number of asymmetric functions, such as in propylene glycol or dipropylene glycol, have their solubility ranges increased. Indeed, polyester **1**, composed of 80% propylene glycol and 20% dipropylene glycol, has a solubility radius of 13.1, which is above the average.

### Unsaturated Polyesters Properties Prediction by QSPR Method

Manipulations to get Hansen solubility parameters of polyesters are repetitive and time-consuming. Each polyester should be diluted in 40 solvents for 24 h and the solubilization results should be interpreted for each solvent. Similarly, measurement of the glass transition temperature requires a DSC and may take more than 1 h for each polymer. It is therefore very useful to develop an easy method to predict these properties in order to save time. To provide a method without the need for extensive analyzes for determination of the glass transition temperature and Hansen parameters of unsaturated polyesters, it was chosen to rely on the initial experimental molar quantities of the monomers introduced into the reactor to calculate the QSPR input descriptors. In order to obtain the final conversion of the synthesized polyesters, the final acid number was recorded for each synthesis. To keep reliable predictions, this method of determination must therefore be limited to unsaturated polyesters with similar monomers and synthetic conditions to the study. Moreover, an additive method already used in literature methods has been chosen (Stefanis and Panayiotou, 2008; Krevelen and Nijenhuis, 2009) and each theoretical structure of polyesters as a function of simple functional groups were decomposed (-CH_2_-, -CH_3_, -COO-, -CH_2_ =CH_2_-, -orthophtalic-, etc. …). In order to obtain the number of theoretical functional groups of a polyester, the Carothers equation on the average degree of polymerization of a step polymerization, nature and the quantity of the monomers introduced into the polycondensation reactor were coupled. In a first step, the stoichiometric ratio between the reagents called *r* was calculated between the initial molar amount of carboxylic acid groups on the initial molar amount of alcohol groups provided by the diacids and glycols of the polycondensation reaction. The conversion of the reaction called *p* was calculated by the ratio of the molar amount of carboxylic acids per gram of resin during the reaction to the initial molar amount per gram of resin. This conversion is followed by the acid number of the polycondensation reaction. The final conversion thus corresponds to the remaining amount of carboxylic acids per gram of resin over the initial amount per gram of resin. The average degree of polymerization is obtained thanks to the Carothers Equation (4).

(4) $$\begin{array}{r} DPn_{theo}=\frac{1+r}{1+r-2rp} \end{array}$$

Once the average degree of polymerization is obtained, the polyester chain was divided into three distinct parts, the two terminal diols from one end to the other of the chain, the repeating units (diols + diacids) and finally a diacid unit binding one of the terminal diols with the first diol repeating unit. To simplify the calculation, the ester functions were integrated in the diacid patterns. The formula to calculate the number of theoretical functional groups is given by Equation (5).

(5) $$\begin{array}{l} FG_{theo}=\left(\sum_{i=1}^{n}2.0\times FG_{endgroup-glycol_{i}}\times {\%}_{mol}glycol_{i}\right)\\ +\left(\sum_{i=1}^{n}\frac{\left(DPn_{theo}-3.0\right)}{2}\times FG_{repetition\;unit-glycol_{i}}\times {\%}_{mol}glycol_{i}\right)\\ +\left(\sum_{j=1}^{m}\frac{\left(DPn_{theo}-3.0\right)}{2}\times FG_{repetition\;unit-diacid_{j}}\times {\%}_{mol}diacid_{j}\right)\\ +\left(\sum_{j}^{m}FG_{link-diacid_{j}}\times {\%}_{mol}diacid_{j}\right)+2.0\\ \times \left(100.0-{\%}_{mol}monoalcool\right)\times FG_{OH} \end{array}$$

The value %_*mol*_*glycol*_*i*_ corresponds to the molar part represented by one of the glycols on all the glycols used in the reaction. The value %_*mol*_*diacid*_*i*_ is the equivalent for the diacid part of the synthesis. As an example for the number of functional groups in the diols, the propylene glycol comprises a –CH_3_ group, a -CH_2_- group and a -CH- group. The -OH end-of-chain groups must also be added. If the polycondensation reaction comprises monofunctional alcohols, these must be added to the terminal glycols in proportion to their molar ratios with respect to the total molar quantity of the glycols of the reaction. The addition of mono-alcohols also has an impact on the amount of alcohol functional groups at the end of the chain. As regards the diacids, itaconic acid comprises for example two -COO- groups, a -CH=CH_2_ group and a -CH_2_- group. The list of functional groups according to the different theoretical structures of the synthesized unsaturated polyesters is given in Table S2.

#### Hansen Solubility Parameter Prediction by QSPR Method

As for the determination of the glass transition temperature, a QSPR method was also applied for the prediction of the δ_*d*_, δ_*p*_, and δ_*h*_ components of the Hansen solubility parameters. The values of the coefficients of the functional groups obtained by the QSPR method are listed in Table 3.

**Table 3:** Coefficients of the linear regression for HSP prediction.

**Functional Group**	***δ_*d*_***	***δ_*p*_***	***δ_*h*_***	***Ra***
-CH_3_	12.8	−22.4	26.5	−21.5
-CH_2_-	0.4	−0.25	0.7	−0.2
-CH-	−26.0	44.56	−52.9	42.8
-C-	−39.2	67.4	−80.5	64.9
-Cyclohexane-	37.7	−67.4	77.4	−64.36
-CH = CH-	0.4	−0.83	1.2	−1.1
-CH = CH_2_	0.2	−0.79	0.95	−1.1
-O-	12.8	−21.7	25.1	−20.8
-COO-	6	−10.4	11.8	−10.0
-OH	21.8	−34.8	43.2	−32.9
-Ortho-	1.5	−1.0	3.2	−1.0

The coefficients obtained for the δ_*h*_ prediction of unsaturated polyesters confirm the hypotheses depicted in section Unsaturated polyesters properties prediction by QSPR method. Indeed, each -CH- and -C- group within the polyester chain, respectively, decreases the δ_*h*_ of −52.9 and −80.5. These groups decrease the linearity of the polyester chains and inhibit the creation of hydrogen bonds between the chains. On the other hand, the other groups such as -CH_3_, -cyclohexane-, -OH, and -O- are the groups which bring the most regularity to the polyester chains and thus increase the creation of polyester bonds.

Unlike the Stephanis-panayiotou or Hoftyzer-Van Krevelen methods, the QSPR method effectively predicts whether a polyester can be soluble in a wide range of solvents or not *via* the determination of *R*_0_. This possibility of prediction is critical in the industrial world in order to save handling time and to be able to quickly develop new resins. Indeed, it will be possible to know in advance the solubility or otherwise of an unsaturated polyester in a new solvent whose Hansen parameters are known. The influence of each functional group on the solubility radius of the unsaturated polyesters is obtained by means of the coefficients of the multiple linear equation. The groups -CH- and -C- have positive coefficients, respectively, of 42.8 and 64.9. They therefore have a positive influence on the solubility radius and allow solubilization of the polyesters in more solvents. As stated in section Unsaturated Polyesters Properties Prediction by QSPR Method, these groups introduce rigidity and asymmetries into the polyester chain. This prevents the polyester chains from associating and favors the polymer-solvent bonds. On the contrary, the -cyclohexane-, -CH_2_-, and -CH_3_- type units favor the association of the chains by their regularity. The groups -O-, -COO-, and -OH are groups allowing the hydrogen bonds. When the polyester is solubilized in a solvent which does not have the capacity to form hydrogen bonds, the polyester will therefore tend to form these hydrogen bonds interchain way and thus promote the association and non-solubilization.

Two techniques described in the literature on the prediction of Hansen solubility parameters of polymers, namely the Hoftyzer—Van Krevelen (Krevelen and Nijenhuis, 2009) and Stefanis—Panayiotou (Stefanis and Panayiotou, 2008) methods, allow to obtain the coefficient of each functional group to use them next in a multilinear equation. The division of the structure of the synthesized polyesters into simple functional groups has been resumed to perform the parameters calculation for the three methods. The comparison of the mean absolute error (MAE) and correlation coefficient R^2^ of the calculation compared to the experimental values of these three methods is made in Table 4.

**Table 4:** Comparison of the MAE and correlation coefficient *R*^2^ for the three methods of HSP prediction.

	**δ_*d*_**		**δ_*p*_**		**δ_*h*_**	
**Methods**	**MAE**	***R*^**2**^**	**MAE**	***R*^**2**^**	**MAE**	***R*^**2**^**
Hoftyzer—Van Krevelen	0.7	0.08	10.2	0.00	5.5	0.49
Stephanis—Panayiotou	0.7	0.00	1.9	0.74	1.1	0.89
QSPR method (This work)	0.5	0.55	0.3	0.96	0.4	0.85

The MAE of the three prediction methods for δ_*d*_ are almost equivalent. The QSPR method adapted to unsaturated polyesters therefore has a limited interest on this parameter. However, correlation coefficient for δ_*d*_is much better for the QSPR method. On the other hand, the QSPR method has a much lower absolute error on the δ_*p*_ parameter than the two other methods described in the literature as well as a better correlation coefficient than the methods found in literature. Mean absolute error for δ_*h*_ is the lowest with QSPR method but Stephanis-Panayiotou method has a slightly better R^2^ for δ_*h*_ prediction than the QSPR method. Globally, the QSPR method is more accurate with unsaturated polyester HSP prediction. The prediction method Hoftyzer-Van krevelen is particularly suitable for high molecular weight polymers of different natures which is not the case for oligomeric unsaturated polyesters. The Stephanis-Panayiotou method is also more reliable for this kind of polymers. Our QSPR method which has been developed specifically on unsaturated polyester proved to be more reliable than the two other models for prediction of the Hansen solubility parameters of the same polymers.

#### Glass Transition Temperature Prediction by QSPR Method

Methods of predicting the glass transition temperature already exist in the literature (Katritzky et al., 1996; Bicerano, 2002; Krevelen and Nijenhuis, 2009). However, in the same way as for the prediction of Hansen parameters, these are optimal for high molecular weight polymers. Thus, a QSPR method applied to unsaturated polyesters may also be particularly suitable to predict *T*_*g*_. In order to correlate the impact of each functional group on the glass transition temperature of the polyesters, a multiple linear regression is set up again in order to obtain the best coefficient of correlation *R*^2^. The evolution of the correlation coefficient as a function of the functional groups introduced into the equation is described in Table 5.

**Table 5:** Evolution of the correlation coefficient (*R*^2^) depending of the descriptors used for *T*_*g*_ modeling.

**Descriptor(s) used**	***R*^**2**^ prediction vs. experimental**
-Ortho-	0.37
-Ortho-, -CH_3_	0.56
-Ortho-, -CH_3_, -O-	0.67
-Ortho-, -CH_3_-, -O-, -CH-	0.72
-Ortho-, -CH_3_-, -O-, -CH-, -CH_2_-	0.74
-Ortho-, -CH_3_-, -O-, -CH-, -CH_2_-, -C-	0.93

**Figure 1:**
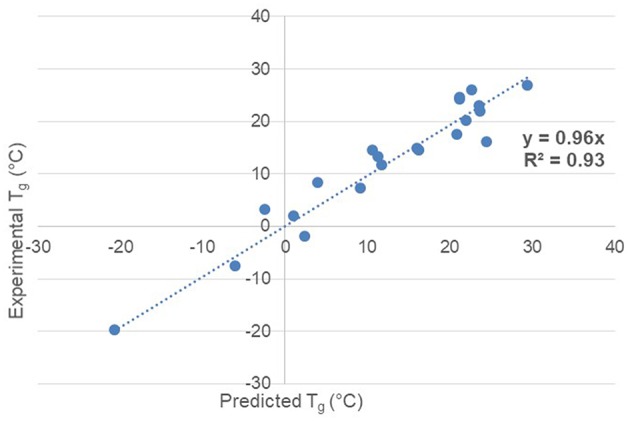
*T*_*g*_ prediction accuracy vs. experimental via QSPR method.

With the six descriptors which are the -Ortho-, -CH_3_-, -O-, -CH-, -CH_2_-, and -C- groups, the prediction of the glass transition temperature of the synthesized unsaturated polyesters is effective (Figure 1) and practical.

The mean absolute error is 2.7°C. The list of coefficients of each descriptor with respect to the regression equation is given in Table 6.

**Table 6:** Coefficients of the linear regression for *T*_*g*_ prediction.

**Descriptor(s)**	**Coefficient**
Intercept	−5.44
-CH_2_-	−4.60
-CH_3_	−4.03
-CH-	11.54
-C-	18.79
-O-	−7.92
-Ortho-	6.91

In addition to provide a linear equation allowing the extrapolation of the glass transition temperature of unsaturated polyesters with structures which are different from those already tested, these coefficients validate the concepts stated in part 3.1. The group -CH_2_- having a coefficient of −4.60, the aliphatic chains such as adipic acid or 2-ethylhexanol do indeed have a plasticizer effect within the polyester chains. It is the same for the ether groups with, for example, dipropylene glycol or diethylene glycol. The introduction of -CH_3_ groups within the polyester also has a negative effect on the glass transition temperature of the polyester (coefficient at −4.03) by the introduction of free volume between the chains. The groups -CH- and -C- by their steric hindrance have a mobility much smaller than a -CH_2_- group or a -CH_3_ group. In the polyester chain, they induce additional rigidity which results in an increase in the glass transition temperature. The same principle also applies when aromatic groups are introduced within the polyester chains. Until now, this prediction model is suitable for unsaturated polyesters with alcohol endings as well as aromatic groups based on orthophthalic anhydride. In fact, polyesters with acid terminations do not have the same hydrogen bonding capacity as the alcohol chain-ends. This difference must certainly play a role in establishing the glass transition temperature of polyesters. On the other hand, the impact on the glass transition temperature of the type of introduced aromatic acid, namely ortho-, iso-, tere-phthalate, within the polyester is significant because of the difference in steric hindrance. This prediction model does not take into account these constraints. These should be studied in a future work.

### Unsaturated Polyester (UP) Viscosity Prediction by QSPR Method

**Figure 2:**
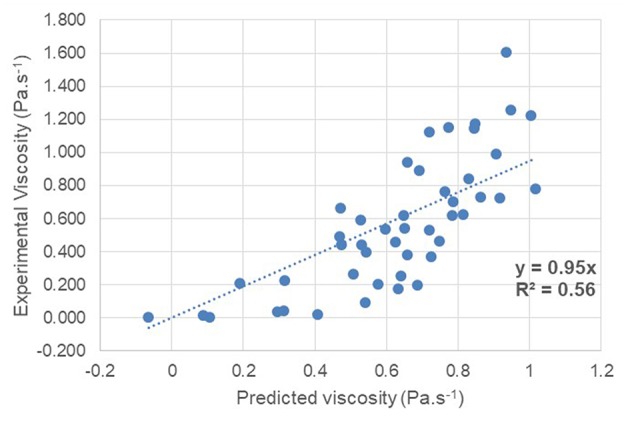
Prediction accuracy of UP viscosity in solution according to the QSPR method.

A QSPR method was also applied to see if it was effective in predicting the viscosity of the unsaturated polyester resins in the database. The input data correspond to the number average molecular weight of the polyester *M*_*n*_ (obtained by SEC), its dispersity index Ð (obtained by SEC), its glass transition temperature *T*_*g*_ (obtained by DSC), the *RED* polymer-solvent compatibility (obtained via HSPiP), the molar volume of the solvent *M*_*vol*_ (obtained via HSPiP), the concentration of the polyester in the solution. The target property of the QSPR method is the measured viscosity of each entry in the database. The coefficients of the multiple linear equation obtained from the 80% of the database were used to predict the viscosities of the remaining 20% of the database. The comparison between predicted and experimental viscosities is shown in Figure 2.

The prediction accuracy of the solution viscosity of polyesters by QSPR is low. The coefficient of correlation *R*^2^ obtained by QSPR is 0.56. The mean absolute error (MAE) is 0.22 Pa.s^−1^. This inefficiency of the prediction is explained by the limitation of the QSPR model to linear phenomena. However, the descriptors used maybe have a non-linear influence. Neural networks are therefore of great interest in this type of application and were tried in the next step.

### Setup of the Neural Network

#### Inputs Selection

In order to set up a neural network allowing the future prediction of unsaturated polyesters viscosities in solution, several descriptors have been chosen as factors having potentially an impact on the viscosity. Seven descriptors were chosen, namely the number average molecular weight *M*_*n*_ (polystyrene equivalent) of the polyester (Ebewele, 2000; Mark, 2007), its dispersity index Ð (Lundberg et al., 1960; Cross, 1969), its glass transition temperature *T*_*g*_ (Young and Lovell, 1996; Ebewele, 2000; Mark, 2007), the polyester-solvent compatibility denoted *RED* (Flory, 1942; Hillyer and Leonard, 1973; Krevelen and Nijenhuis, 2009), the molar volume of the solvent *M*_*vol*_ (Louwerse et al., 2017), the δ_*h*_ of the solvent (Krevelen and Nijenhuis, 2009) and the concentration of the polyester in the resin (Hillyer and Leonard, 1973; Louwerse et al., 2017). This choice was based on the existing literature describing the physical chemistry of polymers. However, it is important to check that these factors really have an impact and that they allow the neural network to build a reliable model based on these factors. In a first step, the impact of each descriptor is tested by calculating the linear correlation coefficients of each descriptor one by one on the measured viscosities (Table 7).

**Table 7:** Linear correlation coefficient *R*^2^ of each descriptor one by one on unsaturated polyester viscosity in solution.

**Descriptor**	***R*^**2**^**
Concentration	0.495
*T_*g*_*	0.383
*M_*n*_*	0.328
M_vol_	0.289
δ_H_	0.097
Ð	0.049
*RED*	0.037

**Figure 3:**
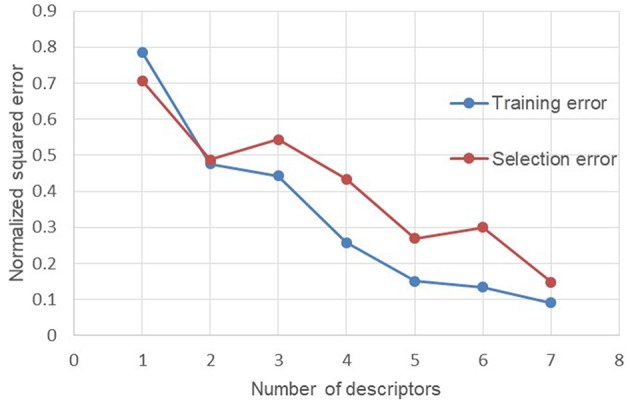
Evolution of the normalized squared error depending of descriptors used for training.

In order to test the quality of each descriptors in the neural network, 80% of the database was used for the training of the neural network and the remaining 20% to test the impact of the number of descriptors used on the normalized squared error obtained between the predicted viscosity and the experimental viscosity. Firstly, the neural network is trained only with the descriptor with the most important linear correlation coefficient *R*^2^ (Table 7). The normalized squared error (NSE) following the training is calculated on both the training and test values. Then the second descriptor with the most important *R*^2^ was added to the first one to see if it reduces the NSE. The third descriptor was then added to see again if the NSE still improve. This procedure was repeated until the integration of all the descriptors of the database. This test proved that there were no useless descriptors or no over-fitting during the test phase of the neural network. The results of these trainings are shown in Figure 3.

**Figure 4:**
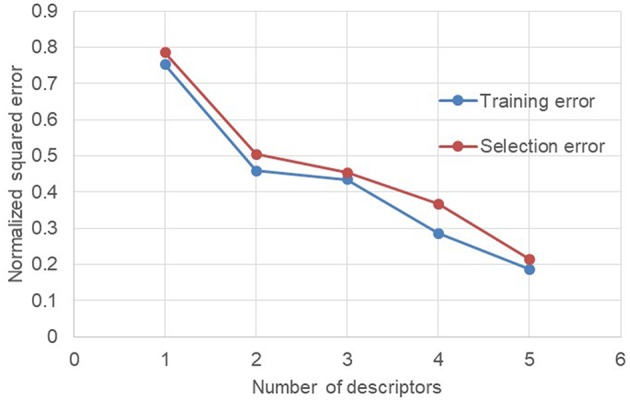
Evolution of the normalized squared error depending of descriptors (without *M*_*n*_ and Ð) used for training.

The evolution of the NSE according to the descriptors added for the training of the neural network makes it possible to see that there are two descriptors which do not improve the performances of the neural network. These two descriptors are the number average molecular weight *M*_*n*_ (descriptor 3) and the dispersity index Ð (descriptor 6). In order to check the performance of the neural network without these two descriptors, a new test was launched only with the remaining five descriptors (Figure 4).

Without the number average molecular weight *M*_*n*_ and the dispersity index Ð, the decrease in NSE is much more regular. In addition, the neural network goes from 7 descriptors in input to only 5 while keeping identical performances. The reduction of the descriptors number is beneficial for the neural network since this may avoid over-fitting phenomena when there are too many descriptors. In addition, from a practical point of view, the limitation of the number of descriptors required allows to set up and enrich an important database by reducing the number of information required for each manipulation.

#### Optimization of the Number of Neurons

**Figure 5:**
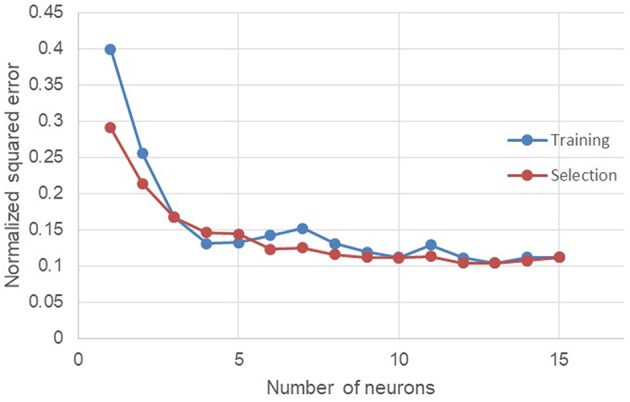
Evolution of the normalized squared error depending of the number of neurons.

The number of neurons in the hidden learning layer is an important parameter to optimize (Díaz-Rodríguez et al., 2014). Indeed, if there are too few neurons in relation to the complexity of the problem, there is a risk of under-fitting due to a lack of parameters. On the other hand, if there are too many neurons hidden in the learning layer, there is a risk of over-fitting during the prediction phase of the target property. In order to have a correct number of learning neurons, the database was randomly divided again with 80% of the inputs intended for learning and 20% for the test. Then the neural network was trained and then tested with a growing number of learning neurons. Three training and selection tests per number of neurons were performed to obtain the lowest normalized squared errors. The results are shown in Figure 5.

Between 0 and 3 learning neurons, under-fitting problems occur because the errors found are the highest in the range of the number of neurons tested. As the number of neurons increases, the errors decrease until they become stable. Similar tests have been conducted up to 40 hidden learning neurons without errors in the learning or testing phases indicating the occurrence of an over-fitting phenomenon. However, the multiplication of the number of neurons also implies the increase of the number of calculations and therefore a greater need for computation needs. As part of this work, the number of neurons was set at 12.

#### Training of the Neural Network

**Figure 6:**
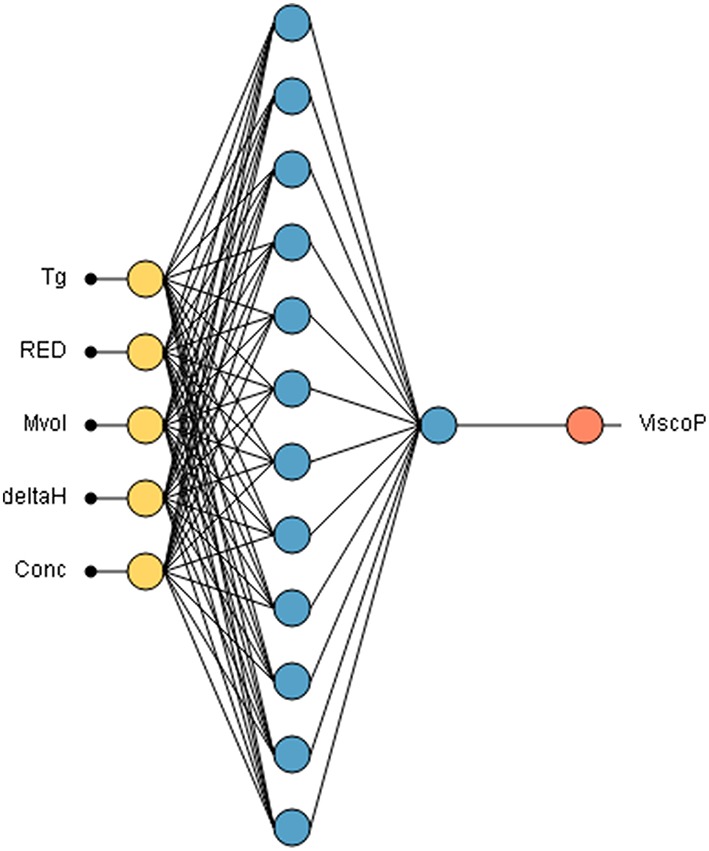
Neural network used for unsaturated polyester resin viscosity prediction Input data are introduced through yellow neurons, the 12 learning neurons are represented in blue. One neuron in a second layer sum up linearly the outputs of the first layer. The orange neuron is the viscosity output neuron.

The neural network was trained with 80% randomly selected from the database created. The neural network is composed of 5 inputs, namely the glass transition temperature *T*_*g*_ of the polyester, the *RED* (polymer-solvent compatibility), the δ_*h*_ of the solvent, its molar volume *M*_*vol*_ and the concentration of the polyester in the solvent. The hidden learning layer has 12 neurons and consequently 85 synapses. The neural network used in this study is illustrated in Figure 6.

The activations functions used are the hyperbolic tangents. The training algorithm chosen is the quasi-Newton method (Setiono and Hui, 1995) with a normalized squared error. This algorithm is based on Newton's method but does not require the computation of the second derivative to find the local minimum of the error. Instead, the quasi-newton method computes an approximation of the inverse Hessian matrix at each iteration of the algorithm, by only using gradient information. A regularization coefficient of 0.01 was applied in order to have a better generalization of the model.

### Influence of Each Descriptor on Viscosity

**Figure 7:**
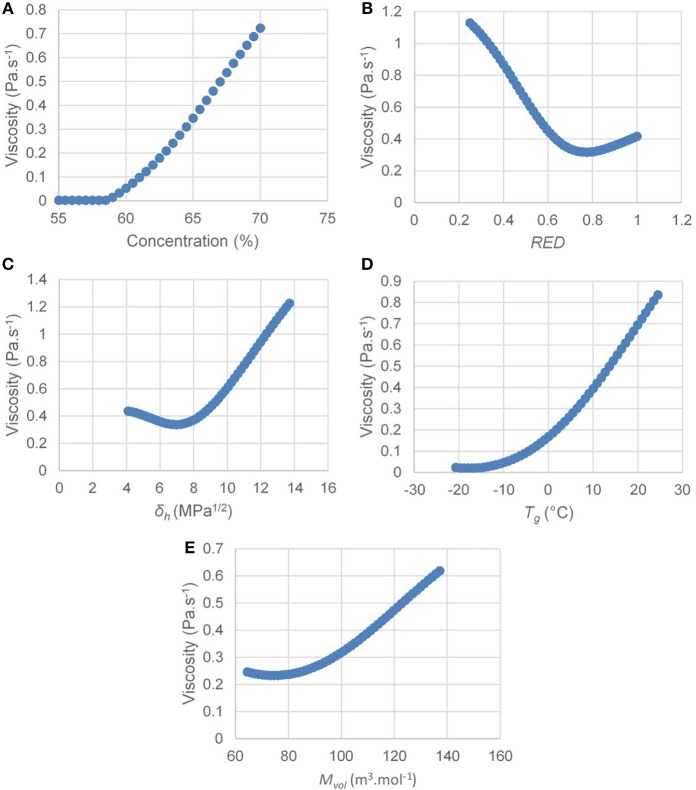
Influence of each descriptor used in the neural network on the unsaturated polyester viscosity in solution [**(A)** influence of concentration; **(B**) influence of *RED*; **(C)** influence of δ_*h*_; **(D)** influence of *T*_*g*_; **(E)** influence of *M*_*vol*_].

Once the neural network is trained, it is possible to isolate the influence of each descriptor on the viscosity by fixing the others by their average. This provides valuable information for understanding the phenomena influencing unsaturated polyester viscosity in concentrated solution. The results are shown in Figure 7.

The evolution of the viscosity as a function of the polyester concentration in the solution is represented by Figure 7A. This model obtained via the neural network corresponds to the models conventionally described in the literature (Yang, 1996). Indeed, taking into account other fixed descriptors, the viscosity of the polyester in solution slowly changes to 58.5% by weight of the polyester and the slope increases substantially thereafter. This phenomenon is due to the overrun of the critical concentration of the polyester in a solvent (Takahashi et al., 1985). At a concentration below the critical concentration, the number of chain entanglements of polymers is low with respect to concentration. While this number of entanglements increases drastically above the maximum critical concentration which causes the increase in the viscosity slope after 58.5% by weight of polyester in the solution.

The influence of polymer-solvent compatibility (*RED*) on viscosity is shown in Figure 7B. The viscosity of the polyester decreases progressively when the *RED* goes from 0.2 to 0.7 and then increases again from 0.7 to 1. This evolution of the viscosity can be explained from the point of view of the hydrodynamic volume occupied by the polymer in solution. When it is a dilute solution of polymer, the more it will be compatible with its solvent, the higher its hydrodynamic volume will be. Indeed, the number of polymer-solvent interactions being de facto high, the polymer chains will be relaxed. The entanglements of chains in the solution will therefore be maximized and the viscosity of the polymer in solution will increase. On the contrary, if the solvent is very poor compatible with the polymer, it will minimize these interactions with the solvent. It will shrink in the form of a globule, reduce its hydrodynamic volume, generate less entombment and thus reduce the viscosity in solution (Hillyer and Leonard, 1973). It is this phenomenon which explains the decrease of the viscosity for the *RED* from 0.2 to 0.7. However, in the case of unsaturated polyester resins, the polymer concentrations are high. When the solvent become incompatible, the globule-like polymer chains will agglomerate to further minimize interactions with the solvent. This agglomerate of globule therefore has a larger hydrodynamic volume than the isolated globule, which implies a slight increase in viscosity from 0.7 in *RED* up to 1. This phenomenon has already been described in the literature (Burrell, 1973; Hillyer and Leonard, 1973) but the use of a neural network allows to find this result thanks to the processing of the data obtained.

Regarding the influence of δ_*h*_ on the polyester viscosity in solution represented in Figure 7C, the viscosity decreases between 4.1 and 7.0 MPa^1/2^ and then increases significantly between 7.0 and 13.7 MPa^1/2^. This phenomenon has already been described in the literature by Nelson who has taken over the classification of solvents from Pimentel and McClellan (Burrell, 1973). The solvents are classified in four categories namely: (a) proton donors (chloroform for example), (b) proton acceptors (ketones, esters, ethers, aromatic hydrocarbons for example), (c) proton donors and acceptors (alcohols, carboxylic acids, water for example), and (d) absence of hydrogen bonds (such as aliphatic hydrocarbons). The solvents used in the database of polyesters in solution with δ_*h*_ values between 4.1 and 7.0 MPa^1/2^ are in category (b) some non-exhaustive examplesof which are styrene (δ_*h*_ = 4.1 MPa^1/2^), cyclohexanone (δ_*h*_ = 5.1 MPa^1/2^), methyl methacrylate (δ_*h*_ = 5.4 MPa^1/2^), acetone (δ_*h*_ = 7.0 MPa^1/2^). Since the polyesters are acceptors and donors of hydrogen bonds (terminal alcohol functions and ester functions), the proton acceptor solvents allow the hydrogen bonds between the polyester chains to be broken. The slight decrease in the viscosity between 4.1 and 7.0 MPa^1/2^ is due to the greater capacity of solvents such as ketones, esters or ethers (δ_*h*_ = 5.0–7.0 MPa^1/2^) to accept hydrogen bonds with respect to typical solvents such as aromatic hydrocarbons (δ_*h*_ = 4.0–5.0 MPa^1/2^). On the contrary, the solvents possessing the higher δ_*h*_ belong to category (c) and are both acceptors and proton donors (acetic acid δ_*h*_ = 13.5 MPa^1/2^, benzyl alcohol δ_*h*_ = 13.7 MPa^1/2^). Before they can break the established hydrogen bonds between the polyester chains, the solvents with high δ_*h*_ must first break their own hydrogen bonds. This phenomenon leads for the polyester a longer and harder dissolution in these kind of solvents. In addition, there is also formation of a denser network of hydrogen bonds between the polyester chains and the solvent molecules. This network is at the origin of the drastic increase in viscosity for solvents with δ_*h*_ between 7 and 14 MPa^1/2^.

The glass transition temperature of the polyester also influences the viscosity of the unsaturated polyester in solution (Figure 7D). Indeed, the higher the glass transition temperature (constant molecular weight), the higher the viscosity. The glass transition temperature is directly related to the rigidity of the chain. Thus, when the polyester chains are in solution at high concentration, the energy required for the mobility of the rigid chains will be greater compared to flexible chains. Rigid chains will therefore have a higher viscosity with respect to these flexible chains (Berry and Fox, 1968).

Regarding the influence of the molar volume of the solvent (Figure 7E), the viscosity increases as the molar volume of the solvent increases (Flory, 1942; Louwerse et al., 2017). This evolution can be explained by the entropy of mixing (solvent + polymer) (Equation 6).

(6) $$\begin{array}{r} \Delta S_{mix}=\;-R\times \left(xlnx+\left(1-x\right)\ln\left(1-x\right)\right) \end{array}$$

The value *x* is the molar fraction of the polymer and *R* is the ideal gas constant. Solvents with small molar volumes give a greater entropy of mixture per liter of solvent. They are therefore better solvents.

### Prediction of Unsaturated Polyester Viscosity in Solution With Neural Network

**Figure 8:**
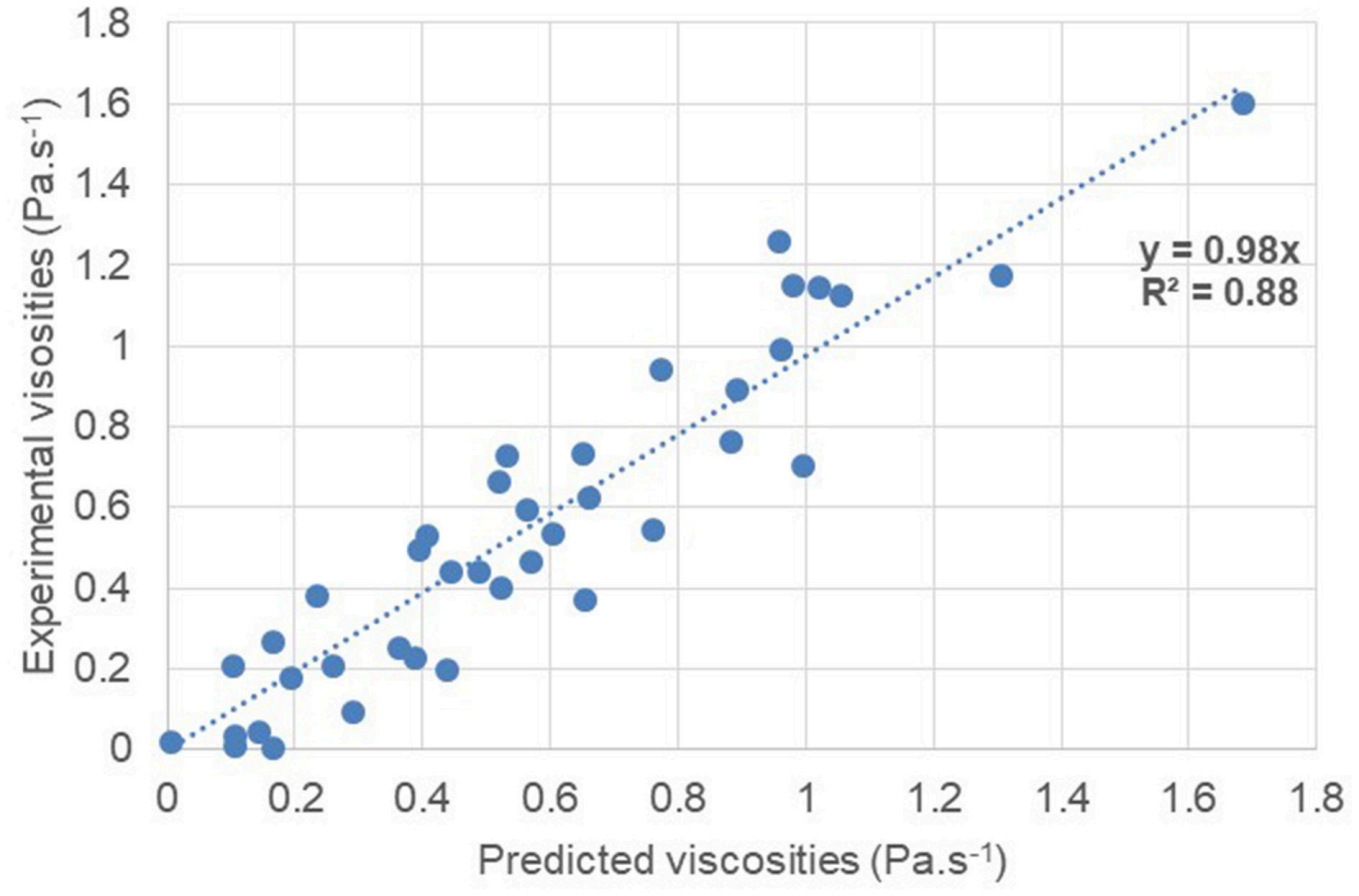
Prediction accuracy of UP viscosity in solution according to the trained Neural Network.

In order to compare the prediction efficiency of the neural network with the QSPR method, the neural network was trained with the same 80% of the database used for the QSPR method. The remaining 20% of the database was tested to compare the predicted viscosity with the experimental viscosity. The prediction accuracy of the trained neural network is represented in Figure 8.

A correlation coefficient *R*^2^ = 0.88 was obtained thanks to the trained neural network. The mean absolute error is 0.115 Pa.s^−1^. The prediction efficiency is much higher with the neural network compared to the QSPR method. This method is therefore particularly suitable for this type of application.

#### K-Fold Cross-Validation

K-Fold cross-validation is a method of validating the neural network to determine predictability. Indeed, all entries in the database are used to check the model. The database is divided into K fractions. In this work, the database was divided into 5 fractions (*K* = 5). The neural network was initially trained with 4 fractions of the database. The fifth fraction, which was not used for training, was used for the neural network prediction test. This operation was repeated 5 times with a different K fraction each time for the test phase. The averages of the correlation coefficients *R*^2^ and the mean average error (MAE) obtained are given in Table 8.

**Table 8:** Results of the K-fold cross validation (*K* = 5) method for the viscosities prediction.

**Viscosities range (Pa.s^**−1**^)**	***R^2^***	**MAE (Pa.s^**−1**^)**
0.003–1.889	0.85	0.116

The *R*^2^ and MAE values obtained by the K-fold cross validation method allow the validation of the neural network stability as well as its ability to effectively predict the viscosity of unsaturated polyester resins. The current database includes 220 entries divided between 179 entries for training and 41 entries for testing the trained neural network. The latter has already shown to be very effective compared to a QSPR model. It might be interesting to extend this comparison by expanding the database. To do this, other polyester resins can be synthesized to teach the neural network new structures and new solvents can also be added.

## Conclusion

The viscosity of unsaturated polyester resins is a very important criterion in the industrial field. Indeed, a viscosity out of specifications can interfere with the handling of the resin and make it impossible to process. This viscosity depends on the chemical structure of the polyester, the nature of the solvent and the concentration of the polyester in solution. The great diversity of existing diols and diacids as well as the current growth of the number of reactive diluents therefore implies a variation of the viscosity which is extremely difficult to predict simply by mathematical or physical laws.

Firstly, in order to avoid experimental input descriptors and to be able to predict the viscosity of polyester resins from theoretical and easily accessible values, a QSPR method has been applied to predict Hansen parameters as well as temperature of glass transition of unsaturated polyesters. This method has proved to be particularly effective compared to other existing methods in the literature because these described methods are based on high molecular weight polymers. However, the QSPR method has proved ineffective for predicting the viscosity of unsaturated polyesters in solution. A classical linear prediction method does not allow non-linear phenomena to be taken into account. It is therefore wise to use machine learning tools.

In this work, a neural network has been set up to verify the ability of such a machine learning process to predict the viscosity of these resins from 21 unsaturated polyesters and 220 mixtures with solvents. This network composed of five descriptors and 12 learning neurons allowed the successful prediction of the viscosity of 41 test resins with an *R*^2^ correlation coefficient of 0.88 and an MAE of 0.116 Pa.s^−1^. These results are very promising given the amount of data available to date. The regular update of the database with the manipulations carried out over time will undoubtedly allow the improvement of the prediction.

## Data Availability

All datasets generated for this study are included in the manuscript and/or the Supplementary Files.

## Author Contributions

JM wrote the manuscript. JM and AL worked on the Neural Network optimization and testing. JM and AL did the polyester syntheses and manipulations. J-VR designed the input descriptors for the neural network. A-SS and CR supervised the study and revised the manuscript.

### Conflict of Interest Statement

The authors declare that the research was conducted in the absence of any commercial or financial relationships that could be construed as a potential conflict of interest.
