# Supplementary material for: Neural Networks Are Promising Tools for the Prediction of the Viscosity of Unsaturated Polyester Resins
Source: Front Chem. 2019 May 27;7:375. doi: 10.3389/fchem.2019.00375 (PMC6545879; doi:10.3389/fchem.2019.00375)
Supplement: Supplementary file 1 [file Table_1.DOCX]

# Neural networks are promising tools for the prediction of the viscosity of unsaturated polyester resins

Julien Molina^1,2*^, Aurélie Laroche^1,2^, Jean-Victor Richard^1^, Anne-Sophie Schuller^1^, Christian Rolando^2^

^1^ Mäder Research, 130, Rue de la Mer Rouge, 68200 Mulhouse, France

^2^ Université de Lille, Faculté des Sciences et Technologies, USR 3290 MSAP, Miniaturisation pour l'Analyse, la Synthèse et la Protéomique, 59655 Villeneuve d'Ascq Cedex, France

TABLE S1 | Unsaturated polyester viscosities in solution database

| **Entry** | **Polyester** | **M_n_ (g.mol^-1^)** | **T_g_ (°C)** | **Solvent** | **RED** | **M_vol_ (m^3^.mol^-1^)** | **δ_h_ (MPa^1/2^)** | **Conc (%wt)** | **ViscoP (Pa.s^-1^)** |
| --- | --- | --- | --- | --- | --- | --- | --- | --- | --- |
| **1** | 1 | 1880.00 | 3.90 | Styrene | 0.91 | 115.70 | 4.10 | 45.00 | 0.03 |
| **2** | 1 | 1880.00 | 3.90 | Styrene | 0.91 | 115.70 | 4.10 | 65.00 | 0.55 |
| **3** | 1 | 1880.00 | 3.90 | Cyclohexanone | 0.41 | 104.20 | 5.10 | 60.00 | 0.60 |
| **4** | 1 | 1880.00 | 3.90 | Methyl Methacrylate | 0.63 | 106.70 | 5.40 | 70.00 | 0.73 |
| **5** | 1 | 1880.00 | 3.90 | n-Butyl Acetate | 0.94 | 132.60 | 6.30 | 62.00 | 0.21 |
| **6** | 1 | 1880.00 | 3.90 | n-Butyl Acetate | 0.94 | 132.60 | 6.30 | 67.00 | 0.51 |
| **7** | 1 | 1880.00 | 3.90 | Acetone | 0.53 | 73.80 | 7.00 | 45.00 | 0.01 |
| **8** | 1 | 1880.00 | 3.90 | Acetone | 0.53 | 73.90 | 7.00 | 53.00 | 0.02 |
| **9** | 1 | 1880.00 | 3.90 | Acetone | 0.53 | 73.80 | 7.00 | 60.00 | 0.09 |
| **10** | 1 | 1880.00 | 3.90 | Acetone | 0.53 | 73.90 | 7.00 | 65.00 | 0.20 |
| **11** | 1 | 1880.00 | 3.90 | Methylene Dichloride | 0.55 | 64.40 | 7.10 | 45.00 | 0.17 |
| **12** | 1 | 1880.00 | 3.90 | Ethyl Acetate | 0.80 | 98.60 | 7.20 | 45.00 | 0.01 |
| **13** | 1 | 1880.00 | 3.90 | Ethyl Acetate | 0.79 | 98.60 | 7.20 | 53.00 | 0.03 |
| **14** | 1 | 1880.00 | 3.90 | Ethyl Acetate | 0.80 | 98.60 | 7.20 | 60.00 | 0.13 |
| **15** | 1 | 1880.00 | 3.90 | Ethyl Acetate | 0.79 | 98.60 | 7.20 | 65.00 | 0.34 |
| **16** | 1 | 1880.00 | 3.90 | γ-Butyrolactone | 0.34 | 76.50 | 7.40 | 60.00 | 0.55 |
| **17** | 1 | 1880.00 | 3.90 | γ-Butyrolactone | 0.34 | 76.50 | 7.40 | 62.00 | 0.55 |
| **18** | 1 | 1880.00 | 3.90 | Tetrahydrofuran | 0.71 | 81.90 | 8.00 | 45.00 | 0.03 |
| **19** | 1 | 1880.00 | 3.90 | Tetrahydrofuran | 0.71 | 81.90 | 8.00 | 53.00 | 0.04 |
| **20** | 1 | 1880.00 | 3.90 | Tetrahydrofuran | 0.71 | 81.90 | 8.00 | 60.00 | 0.19 |
| **21** | 1 | 1880.00 | 3.90 | Propylene Glycol Monomethyl Ether Acetate | 0.88 | 137.10 | 9.80 | 60.00 | 0.27 |
| **22** | 1 | 1880.00 | 3.90 | Propylene Glycol Monomethyl Ether Acetate | 0.88 | 137.10 | 9.80 | 62.00 | 0.53 |
| **23** | 1 | 1880.00 | 3.90 | Propylene Glycol Monomethyl Ether | 0.80 | 98.20 | 11.60 | 60.00 | 0.28 |
| **24** | 1 | 1880.00 | 3.90 | Propylene Glycol Monomethyl Ether | 0.80 | 98.20 | 11.60 | 62.00 | 0.38 |
| **25** | 1 | 1880.00 | 3.90 | Benzyl Alcohol | 0.89 | 103.80 | 13.70 | 45.00 | 0.16 |
| **26** | 1 | 1880.00 | 3.90 | Benzyl Alcohol | 0.53 | 103.80 | 13.70 | 60.00 | 0.84 |
| **27** | 3 | 1560.00 | 16.30 | Styrene | 1.00 | 115.70 | 4.10 | 45.00 | 0.02 |
| **28** | 3 | 1560.00 | 16.30 | Cyclohexanone | 0.41 | 104.20 | 5.10 | 60.00 | 1.12 |
| **29** | 3 | 1560.00 | 16.30 | Methyl Methacrylate | 0.64 | 106.70 | 5.40 | 53.00 | 0.04 |
| **30** | 3 | 1560.00 | 16.30 | Methyl Methacrylate | 0.64 | 106.70 | 5.40 | 65.00 | 0.51 |
| **31** | 3 | 1560.00 | 16.30 | Methyl Methacrylate | 0.64 | 106.70 | 5.40 | 70.00 | 1.10 |
| **32** | 3 | 1560.00 | 16.30 | n-Butyl Acetate | 0.84 | 132.60 | 6.30 | 65.00 | 0.50 |
| **33** | 3 | 1560.00 | 16.30 | n-Butyl Acetate | 0.84 | 132.60 | 6.30 | 70.00 | 1.06 |
| **34** | 3 | 1560.00 | 16.30 | Acetone | 0.47 | 73.80 | 7.00 | 45.00 | 0.01 |
| **35** | 3 | 1560.00 | 16.30 | Acetone | 0.47 | 73.90 | 7.00 | 53.00 | 0.02 |
| **36** | 3 | 1560.00 | 16.30 | Methylene Dichloride | 0.55 | 64.40 | 7.10 | 45.00 | 0.16 |
| **37** | 3 | 1560.00 | 16.30 | Methylene Dichloride | 0.55 | 64.40 | 7.10 | 60.00 | 0.37 |
| **38** | 3 | 1560.00 | 16.30 | Ethyl Acetate | 0.75 | 98.60 | 7.20 | 45.00 | 0.01 |
| **39** | 3 | 1560.00 | 16.30 | Ethyl Acetate | 0.75 | 98.60 | 7.20 | 45.00 | 0.21 |
| **40** | 3 | 1560.00 | 16.30 | Ethyl Acetate | 0.75 | 98.60 | 7.20 | 65.00 | 0.36 |
| **41** | 3 | 1560.00 | 16.30 | γ-Butyrolactone | 0.34 | 76.50 | 7.40 | 60.00 | 0.69 |
| **42** | 3 | 1560.00 | 16.30 | γ-Butyrolactone | 0.34 | 76.50 | 7.40 | 62.00 | 1.07 |
| **43** | 3 | 1560.00 | 16.30 | Tetrahydrofuran | 0.58 | 81.90 | 8.00 | 45.00 | 0.05 |
| **44** | 3 | 1560.00 | 16.30 | Tetrahydrofuran | 0.58 | 81.90 | 8.00 | 60.00 | 0.21 |
| **45** | 3 | 1560.00 | 16.30 | Tetrahydrofuran | 0.58 | 81.90 | 8.00 | 65.00 | 0.46 |
| **46** | 3 | 1560.00 | 16.30 | Propylene Glycol Monomethyl Ether Acetate | 0.87 | 137.10 | 9.80 | 60.00 | 0.63 |
| **47** | 3 | 1560.00 | 16.30 | Propylene Glycol Monomethyl Ether Acetate | 0.83 | 137.10 | 9.80 | 62.00 | 0.62 |
| **48** | 3 | 1560.00 | 16.30 | Propylene Glycol Monomethyl Ether Acetate | 0.83 | 137.10 | 9.80 | 65.00 | 1.32 |
| **49** | 3 | 1560.00 | 16.30 | Propylene Glycol Monomethyl Ether | 0.87 | 98.20 | 11.60 | 62.00 | 0.62 |
| **50** | 3 | 1560.00 | 16.30 | Propylene Glycol Monomethyl Ether | 0.87 | 98.20 | 11.60 | 65.00 | 1.07 |
| **51** | 3 | 1560.00 | 16.30 | Benzyl Alcohol | 0.93 | 103.80 | 13.70 | 45.00 | 0.21 |
| **52** | 4 | 1650.00 | 24.42 | Methyl Methacrylate | 0.74 | 106.70 | 5.40 | 65.00 | 0.87 |
| **53** | 4 | 1650.00 | 24.42 | n-Butyl Acetate | 0.91 | 132.60 | 6.30 | 62.00 | 0.44 |
| **54** | 4 | 1650.00 | 24.42 | n-Butyl Acetate | 0.91 | 132.60 | 6.30 | 65.00 | 0.95 |
| **55** | 4 | 1650.00 | 24.42 | γ-Butyrolactone | 0.25 | 76.50 | 7.40 | 57.00 | 0.87 |
| **56** | 4 | 1650.00 | 24.42 | γ-Butyrolactone | 0.25 | 76.50 | 7.40 | 60.00 | 1.35 |
| **57** | 4 | 1650.00 | 24.42 | Propylene Glycol Monomethyl Ether Acetate | 0.88 | 137.10 | 9.80 | 60.00 | 0.80 |
| **58** | 4 | 1650.00 | 24.42 | Propylene Glycol Monomethyl Ether Acetate | 0.88 | 137.10 | 9.80 | 65.00 | 1.60 |
| **59** | 4 | 1650.00 | 24.42 | Propylene Glycol Monomethyl Ether Acetate | 0.88 | 137.10 | 9.80 | 62.00 | 1.17 |
| **60** | 5 | 1640.00 | 11.66 | Styrene | 0.99 | 115.70 | 4.10 | 65.00 | 0.90 |
| **61** | 5 | 1640.00 | 11.66 | Propylene Carbonate | 0.87 | 85.20 | 4.10 | 62.00 | 0.87 |
| **62** | 5 | 1640.00 | 11.66 | Propylene Carbonate | 0.87 | 85.20 | 4.10 | 60.00 | 0.68 |
| **63** | 5 | 1640.00 | 11.66 | Propylene Carbonate | 0.87 | 85.20 | 4.10 | 65.00 | 0.90 |
| **64** | 5 | 1640.00 | 11.66 | Cyclohexanone | 0.40 | 104.20 | 5.10 | 57.00 | 0.83 |
| **65** | 5 | 1640.00 | 11.66 | Methyl Methacrylate | 0.64 | 106.70 | 5.40 | 65.00 | 0.29 |
| **66** | 5 | 1640.00 | 11.66 | n-Butyl Acetate | 0.84 | 132.60 | 6.30 | 65.00 | 0.46 |
| **67** | 5 | 1640.00 | 11.66 | n-Butyl Acetate | 0.84 | 132.60 | 6.30 | 62.00 | 0.30 |
| **68** | 5 | 1640.00 | 11.66 | n-Butyl Acetate | 0.84 | 132.60 | 6.30 | 67.00 | 0.65 |
| **69** | 5 | 1640.00 | 11.66 | γ-Butyrolactone | 0.34 | 76.50 | 7.40 | 57.00 | 0.52 |
| **70** | 5 | 1640.00 | 11.66 | Propylene Glycol Monomethyl Ether Acetate | 0.83 | 137.10 | 9.80 | 65.00 | 1.14 |
| **71** | 5 | 1640.00 | 11.66 | Propylene Glycol Monomethyl Ether Acetate | 0.83 | 137.10 | 9.80 | 62.00 | 0.57 |
| **72** | 5 | 1640.00 | 11.66 | Propylene Glycol Monomethyl Ether Acetate | 0.83 | 137.10 | 9.80 | 60.00 | 0.36 |
| **73** | 6 | 1780.00 | 16.02 | Styrene | 0.98 | 115.70 | 4.10 | 65.00 | 0.77 |
| **74** | 6 | 1780.00 | 16.02 | Cyclohexanone | 0.40 | 104.20 | 5.10 | 57.00 | 0.89 |
| **75** | 6 | 1780.00 | 16.02 | Methyl Methacrylate | 0.72 | 106.70 | 5.40 | 65.00 | 0.56 |
| **76** | 6 | 1780.00 | 16.02 | n-Butyl Acetate | 0.90 | 132.60 | 6.30 | 65.00 | 0.62 |
| **77** | 6 | 1780.00 | 16.02 | n-Butyl Acetate | 0.90 | 132.60 | 6.30 | 62.00 | 0.35 |
| **78** | 6 | 1780.00 | 16.02 | n-Butyl Acetate | 0.90 | 132.60 | 6.30 | 67.00 | 0.76 |
| **79** | 6 | 1780.00 | 16.02 | γ-Butyrolactone | 0.37 | 76.50 | 7.40 | 60.00 | 0.90 |
| **80** | 6 | 1780.00 | 16.02 | γ-Butyrolactone | 0.37 | 76.50 | 7.40 | 57.00 | 0.60 |
| **81** | 6 | 1780.00 | 16.02 | Propylene Glycol Monomethyl Ether Acetate | 0.86 | 137.10 | 9.80 | 60.00 | 0.61 |
| **82** | 6 | 1780.00 | 16.02 | Propylene Glycol Monomethyl Ether Acetate | 0.86 | 137.10 | 9.80 | 62.00 | 0.81 |
| **83** | 6 | 1780.00 | 16.02 | Propylene Glycol Monomethyl Ether Acetate | 0.86 | 137.10 | 9.80 | 65.00 | 1.25 |
| **84** | 7 | 2528.00 | 21.12 | Styrene | 0.88 | 115.70 | 4.10 | 53.00 | 0.18 |
| **85** | 7 | 2528.00 | 21.12 | Styrene | 0.88 | 115.70 | 4.10 | 60.00 | 0.54 |
| **86** | 7 | 2528.00 | 21.12 | Cyclohexanone | 0.44 | 104.20 | 5.10 | 53.00 | 0.82 |
| **87** | 7 | 2528.00 | 21.12 | n-Butyl Acetate | 0.90 | 132.60 | 6.30 | 62.00 | 0.73 |
| **88** | 7 | 2528.00 | 21.12 | n-Butyl Acetate | 0.90 | 132.60 | 6.30 | 65.00 | 1.22 |
| **89** | 7 | 2528.00 | 21.12 | γ-Butyrolactone | 0.30 | 76.50 | 7.40 | 53.00 | 0.62 |
| **90** | 7 | 2528.00 | 21.12 | γ-Butyrolactone | 0.30 | 76.50 | 7.40 | 57.00 | 1.35 |
| **91** | 7 | 2528.00 | 21.12 | Propylene Glycol Monomethyl Ether Acetate | 0.86 | 137.10 | 9.80 | 53.00 | 0.37 |
| **92** | 7 | 2528.00 | 21.12 | Propylene Glycol Monomethyl Ether Acetate | 0.86 | 137.10 | 9.80 | 60.00 | 1.26 |
| **93** | 7 | 2528.00 | 21.12 | Propylene Glycol Monomethyl Ether Acetate | 0.86 | 137.10 | 9.80 | 65.00 | 1.89 |
| **94** | 7 | 2528.00 | 21.12 | Propylene Glycol Monomethyl Ether | 0.89 | 98.20 | 11.60 | 53.00 | 0.24 |
| **95** | 7 | 2528.00 | 21.12 | Propylene Glycol Monomethyl Ether | 0.89 | 98.20 | 11.60 | 57.00 | 0.56 |
| **96** | 9 | 1610.00 | 21.90 | Styrene | 0.98 | 115.70 | 4.10 | 45.00 | 0.03 |
| **97** | 9 | 1610.00 | 21.90 | Cyclohexanone | 0.45 | 104.20 | 5.10 | 53.00 | 0.41 |
| **98** | 9 | 1610.00 | 21.90 | Cyclohexanone | 0.45 | 104.20 | 5.10 | 60.00 | 1.30 |
| **99** | 9 | 1610.00 | 21.90 | n-Butyl Acetate | 0.83 | 132.60 | 6.30 | 67.00 | 1.38 |
| **100** | 9 | 1610.00 | 21.90 | Acetone | 0.38 | 73.80 | 7.00 | 70.00 | 0.78 |
| **101** | 9 | 1610.00 | 21.90 | Methylene Dichloride | 0.44 | 64.40 | 7.10 | 45.00 | 0.37 |
| **102** | 9 | 1610.00 | 21.90 | Methylene Dichloride | 0.44 | 64.40 | 7.10 | 60.00 | 0.62 |
| **103** | 9 | 1610.00 | 21.90 | Ethyl Acetate | 0.68 | 98.60 | 7.20 | 45.00 | 0.02 |
| **104** | 9 | 1610.00 | 21.90 | Ethyl Acetate | 0.68 | 98.60 | 7.20 | 65.00 | 0.53 |
| **105** | 9 | 1610.00 | 21.90 | Tetrahydrofuran | 0.58 | 81.90 | 8.00 | 45.00 | 0.04 |
| **106** | 9 | 1610.00 | 21.90 | Tetrahydrofuran | 0.58 | 81.90 | 8.00 | 60.00 | 0.32 |
| **107** | 9 | 1610.00 | 21.90 | Tetrahydrofuran | 0.58 | 81.90 | 8.00 | 65.00 | 0.47 |
| **108** | 9 | 1610.00 | 21.90 | Propylene Glycol Monomethyl Ether Acetate | 0.68 | 137.10 | 9.80 | 60.00 | 0.79 |
| **109** | 9 | 1610.00 | 21.90 | Propylene Glycol Monomethyl Ether Acetate | 0.68 | 137.10 | 9.80 | 62.00 | 1.05 |
| **110** | 9 | 1610.00 | 21.90 | Benzyl Alcohol | 0.76 | 103.80 | 13.70 | 45.00 | 0.31 |
| **111** | 12 | 1658.00 | 21.20 | Styrene | 0.89 | 115.70 | 4.10 | 60.00 | 0.47 |
| **112** | 12 | 1956.00 | 21.20 | Styrene | 0.89 | 115.70 | 4.10 | 65.00 | 1.22 |
| **113** | 12 | 1956.00 | 21.20 | Styrene | 0.89 | 115.70 | 4.10 | 53.00 | 0.10 |
| **114** | 12 | 1956.00 | 21.20 | Cyclohexanone | 0.47 | 104.20 | 5.10 | 53.00 | 0.68 |
| **115** | 12 | 1658.00 | 21.20 | n-Butyl Acetate | 0.92 | 132.60 | 6.30 | 62.00 | 0.40 |
| **116** | 12 | 1956.00 | 21.20 | n-Butyl Acetate | 0.92 | 132.60 | 6.30 | 65.00 | 0.98 |
| **117** | 12 | 1956.00 | 21.20 | γ-Butyrolactone | 0.25 | 76.50 | 7.40 | 53.00 | 0.41 |
| **118** | 12 | 1956.00 | 21.20 | Propylene Glycol Monomethyl Ether Acetate | 0.88 | 137.10 | 9.80 | 53.00 | 0.18 |
| **119** | 12 | 1658.00 | 21.20 | Propylene Glycol Monomethyl Ether Acetate | 0.88 | 137.10 | 9.80 | 60.00 | 0.76 |
| **120** | 12 | 1956.00 | 21.20 | Propylene Glycol Monomethyl Ether | 0.90 | 98.20 | 11.60 | 53.00 | 0.27 |
| **121** | 12 | 1658.00 | 21.20 | Propylene Glycol Monomethyl Ether | 0.90 | 98.20 | 11.60 | 57.00 | 0.41 |
| **122** | 14 | 1409.00 | -6.10 | Styrene | 0.99 | 115.70 | 4.10 | 45.00 | 0.02 |
| **123** | 14 | 1409.00 | -6.10 | Cyclohexanone | 0.41 | 104.20 | 5.10 | 60.00 | 0.20 |
| **124** | 14 | 1409.00 | -6.10 | Cyclohexanone | 0.41 | 104.20 | 5.10 | 62.00 | 0.40 |
| **125** | 14 | 1409.00 | -6.10 | Cyclohexanone | 0.41 | 104.20 | 5.10 | 65.00 | 0.82 |
| **126** | 14 | 1409.00 | -6.10 | Methyl Methacrylate | 0.63 | 106.70 | 5.40 | 60.00 | 0.09 |
| **127** | 14 | 1409.00 | -6.10 | Acetone | 0.47 | 73.80 | 7.00 | 45.00 | 0.01 |
| **128** | 14 | 1409.00 | -6.10 | Acetone | 0.47 | 73.80 | 7.00 | 45.00 | 0.12 |
| **129** | 14 | 1409.00 | -6.10 | Ethyl Acetate | 0.75 | 98.60 | 7.20 | 60.00 | 0.06 |
| **130** | 14 | 1409.00 | -6.10 | γ-Butyrolactone | 0.34 | 76.50 | 7.40 | 60.00 | 0.25 |
| **131** | 14 | 1409.00 | -6.10 | γ-Butyrolactone | 0.34 | 76.50 | 7.40 | 65.00 | 0.54 |
| **132** | 14 | 1409.00 | -6.10 | Tetrahydrofuran | 0.69 | 81.90 | 8.00 | 45.00 | 0.03 |
| **133** | 14 | 1409.00 | -6.10 | Tetrahydrofuran | 0.69 | 81.90 | 8.00 | 60.00 | 0.15 |
| **134** | 14 | 1409.00 | -6.10 | Propylene Glycol Monomethyl Ether Acetate | 0.81 | 137.10 | 9.80 | 65.00 | 0.25 |
| **135** | 14 | 1409.00 | -6.10 | Benzyl Alcohol | 0.93 | 103.80 | 13.70 | 60.00 | 0.61 |
| **136** | 15 | 1330.00 | 1.00 | Cyclohexanone | 0.41 | 104.20 | 5.10 | 60.00 | 0.59 |
| **137** | 15 | 1330.00 | 1.00 | Cyclohexanone | 0.41 | 104.20 | 5.10 | 62.00 | 0.55 |
| **138** | 15 | 1330.00 | 1.00 | Methyl Methacrylate | 0.63 | 106.70 | 5.40 | 60.00 | 0.11 |
| **139** | 15 | 1330.00 | 1.00 | Methyl Methacrylate | 0.63 | 106.70 | 5.40 | 65.00 | 0.25 |
| **140** | 15 | 1330.00 | 1.00 | n-Butyl Acetate | 0.84 | 132.60 | 6.30 | 65.00 | 0.32 |
| **141** | 15 | 1330.00 | 1.00 | n-Butyl Acetate | 0.84 | 132.60 | 6.30 | 67.00 | 0.34 |
| **142** | 15 | 1330.00 | 1.00 | Ethyl Acetate | 0.75 | 98.60 | 7.20 | 60.00 | 0.12 |
| **143** | 15 | 1330.00 | 1.00 | Ethyl Acetate | 0.75 | 98.60 | 7.20 | 65.00 | 0.24 |
| **144** | 15 | 1330.00 | 1.00 | γ-Butyrolactone | 0.34 | 76.50 | 7.40 | 60.00 | 0.37 |
| **145** | 15 | 1330.00 | 1.00 | γ-Butyrolactone | 0.34 | 76.50 | 7.40 | 62.00 | 0.50 |
| **146** | 15 | 1330.00 | 1.00 | γ-Butyrolactone | 0.34 | 76.50 | 7.40 | 65.00 | 0.94 |
| **147** | 15 | 1330.00 | 1.00 | Tetrahydrofuran | 0.69 | 81.90 | 8.00 | 60.00 | 0.13 |
| **148** | 15 | 1330.00 | 1.00 | Tetrahydrofuran | 0.69 | 81.90 | 8.00 | 65.00 | 0.23 |
| **149** | 15 | 1330.00 | 1.00 | Propylene Glycol Monomethyl Ether Acetate | 0.83 | 137.10 | 9.80 | 65.00 | 0.59 |
| **150** | 15 | 1330.00 | 1.00 | Propylene Glycol Monomethyl Ether Acetate | 0.83 | 137.10 | 9.80 | 67.00 | 0.80 |
| **151** | 15 | 1330.00 | 1.00 | Propylene Glycol Monomethyl Ether | 0.87 | 98.20 | 11.60 | 65.00 | 0.41 |
| **152** | 15 | 1330.00 | 1.00 | Propylene Glycol Monomethyl Ether | 0.87 | 98.20 | 11.60 | 67.00 | 0.53 |
| **153** | 15 | 1330.00 | 1.00 | Propylene Glycol Monomethyl Ether | 0.87 | 98.20 | 11.60 | 70.00 | 0.59 |
| **154** | 15 | 1330.00 | 1.00 | Benzyl Alcohol | 0.94 | 103.80 | 13.70 | 60.00 | 0.71 |
| **155** | 16 | 950.00 | -2.50 | Styrene | 0.99 | 115.70 | 4.10 | 45.00 | 0.01 |
| **156** | 16 | 950.00 | -2.50 | Styrene | 0.99 | 115.70 | 4.10 | 65.00 | 0.07 |
| **157** | 16 | 950.00 | -2.50 | Cyclohexanone | 0.34 | 104.20 | 5.10 | 60.00 | 0.36 |
| **158** | 16 | 950.00 | -2.50 | Cyclohexanone | 0.34 | 104.20 | 5.10 | 62.00 | 0.75 |
| **159** | 16 | 950.00 | -2.50 | Methyl Methacrylate | 0.53 | 106.70 | 5.40 | 45.00 | 0.01 |
| **160** | 16 | 950.00 | -2.50 | Methyl Methacrylate | 0.53 | 106.70 | 5.40 | 60.00 | 0.06 |
| **161** | 16 | 950.00 | -2.50 | n-Butyl Acetate | 0.74 | 132.60 | 6.30 | 65.00 | 0.18 |
| **162** | 16 | 950.00 | -2.50 | Ethyl Acetate | 0.61 | 98.60 | 7.20 | 45.00 | 0.00 |
| **163** | 16 | 950.00 | -2.50 | Ethyl Acetate | 0.61 | 98.60 | 7.20 | 60.00 | 0.04 |
| **164** | 16 | 950.00 | -2.50 | γ-Butyrolactone | 0.54 | 76.50 | 7.40 | 60.00 | 0.21 |
| **165** | 16 | 950.00 | -2.50 | γ-Butyrolactone | 0.45 | 76.50 | 7.40 | 65.00 | 0.49 |
| **166** | 16 | 950.00 | -2.50 | γ-Butyrolactone | 0.45 | 76.50 | 7.40 | 67.00 | 0.60 |
| **167** | 16 | 950.00 | -2.50 | Tetrahydrofuran | 0.53 | 81.90 | 8.00 | 60.00 | 0.04 |
| **168** | 16 | 950.00 | -2.50 | Propylene Glycol Monomethyl Ether Acetate | 0.65 | 137.10 | 9.80 | 67.00 | 0.55 |
| **169** | 16 | 950.00 | -2.50 | Propylene Glycol Monomethyl Ether | 0.61 | 98.20 | 11.60 | 65.00 | 0.38 |
| **170** | 16 | 950.00 | -2.50 | Benzyl Alcohol | 0.78 | 103.80 | 13.70 | 60.00 | 0.23 |
| **171** | 17 | 2090.00 | 22.88 | Cyclohexanone | 0.40 | 104.20 | 5.10 | 55.00 | 1.15 |
| **172** | 17 | 2090.00 | 22.88 | n-Butyl Acetate | 0.84 | 132.60 | 6.30 | 65.00 | 1.26 |
| **173** | 17 | 2090.00 | 22.88 | n-Butyl Acetate | 0.87 | 132.60 | 6.30 | 60.00 | 0.33 |
| **174** | 17 | 2090.00 | 22.88 | n-Butyl Acetate | 0.87 | 132.60 | 6.30 | 62.00 | 0.57 |
| **175** | 17 | 2090.00 | 22.88 | γ-Butyrolactone | 0.33 | 76.50 | 7.40 | 57.00 | 0.96 |
| **176** | 17 | 2090.00 | 22.88 | γ-Butyrolactone | 0.33 | 76.50 | 7.40 | 53.00 | 0.43 |
| **177** | 17 | 2090.00 | 22.88 | Propylene Glycol Monomethyl Ether Acetate | 0.84 | 137.10 | 9.80 | 60.00 | 0.99 |
| **178** | 17 | 2090.00 | 22.88 | Propylene Glycol Monomethyl Ether Acetate | 0.84 | 137.10 | 9.80 | 62.00 | 1.36 |
| **179** | 19 | 1410.00 | 11.20 | Styrene | 0.89 | 115.70 | 4.10 | 60.00 | 0.18 |
| **180** | 19 | 1410.00 | 11.20 | Styrene | 0.89 | 115.70 | 4.10 | 65.00 | 0.39 |
| **181** | 19 | 1410.00 | 11.20 | Methyl Methacrylate | 0.63 | 106.70 | 5.40 | 45.00 | 0.02 |
| **182** | 19 | 1410.00 | 11.20 | Methyl Methacrylate | 0.63 | 106.70 | 5.40 | 60.00 | 0.17 |
| **183** | 19 | 1410.00 | 11.20 | n-Butyl Acetate | 0.85 | 132.60 | 6.30 | 65.00 | 0.32 |
| **184** | 19 | 1410.00 | 11.20 | n-Butyl Acetate | 0.85 | 132.60 | 6.30 | 67.00 | 0.37 |
| **185** | 19 | 1410.00 | 11.20 | Acetone | 0.45 | 73.80 | 7.00 | 45.00 | 0.00 |
| **186** | 19 | 1410.00 | 11.20 | Acetone | 0.45 | 73.80 | 7.00 | 45.00 | 0.14 |
| **187** | 19 | 1410.00 | 11.20 | Acetone | 0.45 | 73.80 | 7.00 | 45.00 | 0.21 |
| **188** | 19 | 1410.00 | 11.20 | Methylene Dichloride | 0.56 | 64.40 | 7.10 | 45.00 | 0.13 |
| **189** | 19 | 1410.00 | 11.20 | Methylene Dichloride | 0.56 | 64.40 | 7.10 | 60.00 | 0.38 |
| **190** | 19 | 1410.00 | 11.20 | Ethyl Acetate | 0.75 | 98.60 | 7.20 | 45.00 | 0.01 |
| **191** | 19 | 1410.00 | 11.20 | Ethyl Acetate | 0.75 | 98.60 | 7.20 | 60.00 | 0.16 |
| **192** | 19 | 1410.00 | 11.20 | Ethyl Acetate | 0.75 | 98.60 | 7.20 | 65.00 | 0.29 |
| **193** | 19 | 1410.00 | 11.20 | γ-Butyrolactone | 0.34 | 76.50 | 7.40 | 60.00 | 0.46 |
| **194** | 19 | 1410.00 | 11.20 | γ-Butyrolactone | 0.34 | 76.50 | 7.40 | 62.00 | 0.82 |
| **195** | 19 | 1410.00 | 11.20 | γ-Butyrolactone | 0.34 | 76.50 | 7.40 | 65.00 | 0.93 |
| **196** | 19 | 1410.00 | 11.20 | Tetrahydrofuran | 0.71 | 81.90 | 8.00 | 60.00 | 0.21 |
| **197** | 19 | 1410.00 | 11.20 | Propylene Glycol Monomethyl Ether Acetate | 0.83 | 137.10 | 9.80 | 65.00 | 0.70 |
| **198** | 19 | 1410.00 | 11.20 | Propylene Glycol Monomethyl Ether | 0.87 | 98.20 | 11.60 | 65.00 | 0.54 |
| **199** | 19 | 1410.00 | 11.20 | Propylene Glycol Monomethyl Ether | 0.87 | 98.20 | 11.60 | 67.00 | 0.84 |
| **200** | 19 | 1410.00 | 11.20 | Benzyl Alcohol | 0.96 | 103.80 | 13.70 | 45.00 | 0.16 |
| **201** | 21 | 1350.00 | -20.70 | Styrene | 0.99 | 115.70 | 4.10 | 60.00 | 0.12 |
| **202** | 21 | 1350.00 | -20.70 | Styrene | 0.99 | 115.70 | 4.10 | 65.00 | 0.35 |
| **203** | 21 | 1350.00 | -20.70 | Cyclohexanone | 0.43 | 104.20 | 5.10 | 60.00 | 0.36 |
| **204** | 21 | 1350.00 | -20.70 | Cyclohexanone | 0.43 | 104.20 | 5.10 | 65.00 | 0.66 |
| **205** | 21 | 1350.00 | -20.70 | Cyclohexanone | 0.43 | 104.20 | 5.10 | 67.00 | 0.79 |
| **206** | 21 | 1350.00 | -20.70 | Methyl Methacrylate | 0.72 | 106.70 | 5.40 | 60.00 | 0.14 |
| **207** | 21 | 1350.00 | -20.70 | Methyl Methacrylate | 0.72 | 106.70 | 5.40 | 65.00 | 0.26 |
| **208** | 21 | 1350.00 | -20.70 | n-Butyl Acetate | 0.90 | 132.60 | 6.30 | 65.00 | 0.15 |
| **209** | 21 | 1350.00 | -20.70 | n-Butyl Acetate | 0.90 | 132.60 | 6.30 | 67.00 | 0.26 |
| **210** | 21 | 1350.00 | -20.70 | Ethyl Acetate | 0.81 | 98.60 | 7.20 | 60.00 | 0.06 |
| **211** | 21 | 1350.00 | -20.70 | γ-Butyrolactone | 0.33 | 76.50 | 7.40 | 60.00 | 0.22 |
| **212** | 21 | 1350.00 | -20.70 | γ-Butyrolactone | 0.33 | 76.50 | 7.40 | 65.00 | 0.44 |
| **213** | 21 | 1350.00 | -20.70 | γ-Butyrolactone | 0.33 | 76.50 | 7.40 | 67.00 | 0.44 |
| **214** | 21 | 1350.00 | -20.70 | Tetrahydrofuran | 0.72 | 81.90 | 8.00 | 60.00 | 0.06 |
| **215** | 21 | 1350.00 | -20.70 | Propylene Glycol Monomethyl Ether Acetate | 0.88 | 137.10 | 9.80 | 65.00 | 0.40 |
| **216** | 21 | 1350.00 | -20.70 | Propylene Glycol Monomethyl Ether Acetate | 0.88 | 137.10 | 9.80 | 67.00 | 0.41 |
| **217** | 21 | 1350.00 | -20.70 | Propylene Glycol Monomethyl Ether Acetate | 0.88 | 137.10 | 9.80 | 70.00 | 0.79 |
| **218** | 21 | 1350.00 | -20.70 | Propylene Glycol Monomethyl Ether | 0.93 | 98.20 | 11.60 | 65.00 | 0.23 |
| **219** | 21 | 1350.00 | -20.70 | Propylene Glycol Monomethyl Ether | 0.93 | 98.20 | 11.60 | 67.00 | 0.32 |
| **220** | 21 | 1350.00 | -20.70 | Benzyl Alcohol | 0.89 | 103.80 | 13.70 | 60.00 | 0.59 |

TABLE S2 | Functional groups of the synthesized unsaturated polyesters

| **Polyester** | **CH_2_** | **CH_3_** | **CH** | **C** | **Cyclohexane** | **COO** | **CH=CH** | **CH_2_=C** | **O** | **Ortho** | **OH** |
| --- | --- | --- | --- | --- | --- | --- | --- | --- | --- | --- | --- |
| **1** | 7.93 | 7.03 | 7.03 | 0.00 | 0.00 | 9.39 | 3.17 | 0.00 | 1.22 | 1.03 | 2.00 |
| **2** | 11.05 | 11.05 | 1.95 | 4.55 | 0.00 | 11.00 | 11.00 | 0.00 | 0.00 | 0.00 | 2.00 |
| **3** | 9.18 | 9.18 | 1.62 | 3.78 | 0.00 | 8.80 | 2.64 | 0.00 | 0.00 | 1.36 | 2.00 |
| **4** | 8.95 | 8.95 | 1.58 | 3.69 | 0.00 | 8.53 | 2.13 | 0.00 | 0.00 | 1.63 | 2.00 |
| **5** | 9.01 | 9.01 | 1.59 | 3.71 | 0.00 | 8.60 | 3.01 | 0.00 | 0.00 | 0.99 | 2.00 |
| **6** | 9.35 | 9.35 | 1.65 | 3.85 | 0.00 | 9.00 | 2.70 | 0.00 | 0.00 | 1.40 | 2.00 |
| **7** | 15.61 | 14.43 | 2.55 | 5.94 | 0.00 | 14.98 | 4.49 | 0.00 | 0.00 | 2.70 | 2.00 |
| **8** | 9.18 | 9.18 | 1.62 | 3.78 | 0.00 | 8.80 | 0.00 | 2.64 | 0.00 | 1.36 | 2.00 |
| **9** | 5.50 | 5.50 | 5.50 | 0.00 | 0.00 | 9.00 | 2.10 | 0.60 | 0.00 | 1.40 | 2.00 |
| **10** | 5.80 | 5.80 | 5.80 | 0.00 | 0.00 | 9.60 | 2.88 | 0.00 | 0.00 | 1.52 | 2.00 |
| **11** | 10.98 | 8.88 | 2.02 | 3.08 | 0.00 | 8.90 | 2.46 | 0.00 | 0.00 | 1.24 | 1.30 |
| **12** | 12.79 | 12.79 | 2.26 | 5.27 | 0.00 | 13.05 | 3.92 | 0.00 | 0.00 | 2.21 | 2.00 |
| **13** | 16.59 | 1.61 | 9.10 | 0.00 | 3.75 | 8.70 | 2.61 | 0.00 | 0.00 | 1.34 | 2.00 |
| **14** | 10.20 | 10.20 | 10.20 | 0.00 | 0.00 | 8.20 | 1.86 | 0.60 | 5.10 | 1.24 | 2.00 |
| **15** | 10.40 | 10.40 | 5.20 | 2.60 | 0.00 | 8.40 | 2.52 | 0.00 | 2.60 | 1.28 | 2.00 |
| **16** | 5.10 | 5.10 | 0.90 | 2.10 | 0.00 | 4.00 | 1.20 | 0.00 | 0.00 | 0.40 | 2.00 |
| **17** | 10.28 | 10.28 | 1.81 | 4.23 | 0.00 | 10.09 | 3.03 | 0.00 | 0.00 | 1.62 | 2.00 |
| **18** | 20.40 | 0.00 | 10.20 | 0.00 | 5.10 | 8.20 | 2.46 | 0.00 | 0.00 | 1.24 | 2.00 |
| **19** | 8.18 | 7.48 | 1.32 | 3.08 | 0.00 | 8.90 | 2.46 | 0.00 | 0.00 | 1.94 | 1.30 |
| **20** | 17.85 | 3.15 | 7.35 | 1.58 | 3.68 | 8.50 | 2.55 | 0.00 | 0.00 | 1.30 | 2.00 |
| **21** | 14.46 | 9.10 | 1.61 | 3.75 | 0.00 | 8.70 | 2.61 | 0.00 | 0.00 | 0.00 | 2.00 |

**
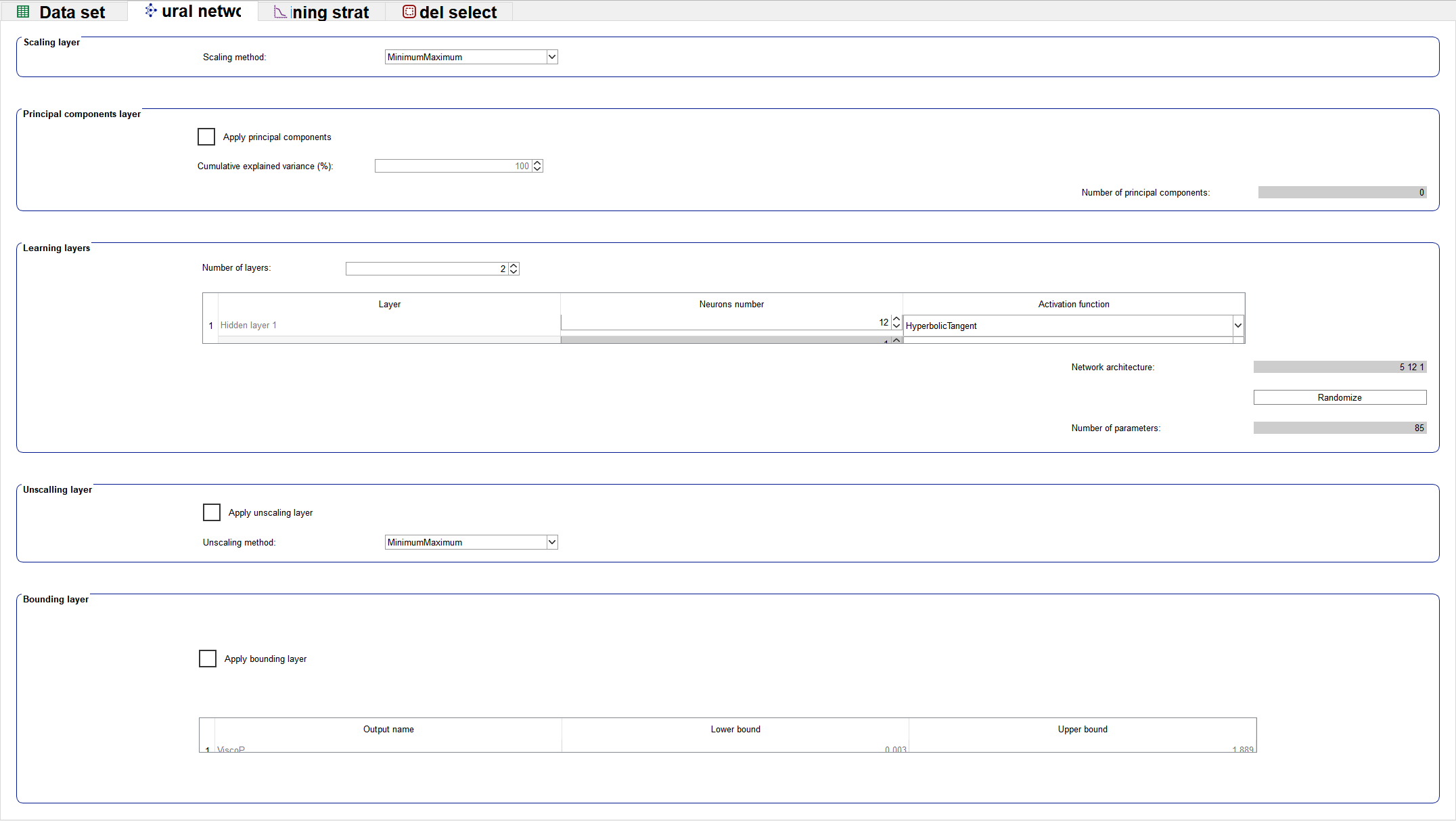
**

FIGURE S3 | Neural Designer software general parameters used for this study


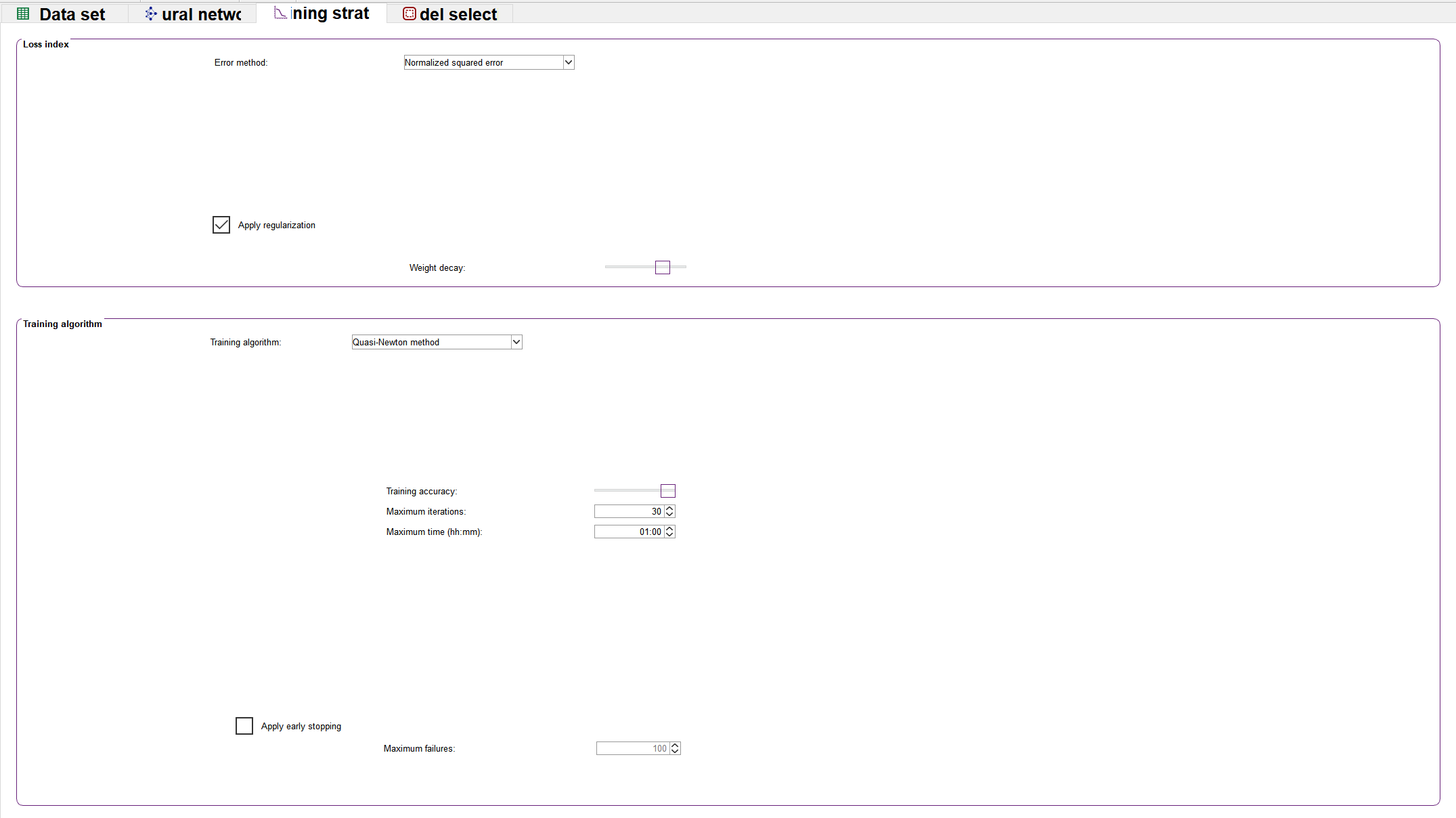


FIGURE S4 | Neural Designer training parameters used for this study
